# UPRLIMET: UPstream Regional LiDAR Model for Extent of Trout in stream networks

**DOI:** 10.1038/s41598-022-23754-0

**Published:** 2022-12-01

**Authors:** Brooke E. Penaluna, Jonathan D. Burnett, Kelly Christiansen, Ivan Arismendi, Sherri L. Johnson, Kitty Griswold, Brett Holycross, Sonja H. Kolstoe

**Affiliations:** 1 U.S. Department of Agriculture, Forest Service, Pacific Northwest Research Station, 3200 SW Jefferson Way, Corvallis, OR 97331 USA; 2 Department of Fisheries, Wildlife, and Conservation Sciences, Oregon State University, 104 Nash Hall, Corvallis, OR 97331 USA; 3 Department of Biological Sciences, Idaho State University, 921 S. 8th Ave Mail, Stop 8007, Pocatello, ID 83209-8007 USA; 4 Pacific States Marine Fisheries Commission, 205 SE Spokane St., Portland, OR 97202 USA; 5 U.S. Department of Agriculture, Forest Service, Pacific Northwest Research Station, 1220 SW 3rd Avenue, Suite 1410, Portland, OR 97204 USA

**Keywords:** Ecological modelling, Freshwater ecology, Population dynamics

## Abstract

Predicting the edges of species distributions is fundamental for species conservation, ecosystem services, and management decisions. In North America, the location of the upstream limit of fish in forested streams receives special attention, because fish-bearing portions of streams have more protections during forest management activities than fishless portions. We present a novel model development and evaluation framework, wherein we compare 26 models to predict upper distribution limits of trout in streams. The models used machine learning, logistic regression, and a sophisticated nested spatial cross-validation routine to evaluate predictive performance while accounting for spatial autocorrelation. The model resulting in the best predictive performance, termed UPstream Regional LiDAR Model for Extent of Trout (UPRLIMET), is a two-stage model that uses a logistic regression algorithm calibrated to observations of Coastal Cutthroat Trout (*Oncorhynchus clarkii clarkii*) occurrence and variables representing hydro-topographic characteristics of the landscape. We predict trout presence along reaches throughout a stream network, and include a stopping rule to identify a discrete upper limit point above which all stream reaches are classified as fishless. Although there is no simple explanation for the upper distribution limit identified in UPRLIMET, four factors, including upstream channel length above the point of uppermost fish, drainage area, slope, and elevation, had highest importance. Across our study region of western Oregon, we found that more of the fish-bearing network is on private lands than on state, US Bureau of Land Mangement (BLM), or USDA Forest Service (USFS) lands, highlighting the importance of using spatially consistent maps across a region and working across land ownerships. Our research underscores the value of using occurrence data to develop simple, but powerful, prediction tools to capture complex ecological processes that contribute to distribution limits of species.

## Introduction

Understanding the edges of a species distribution is fundamental for species conservation, ecosystem services, and management decision-making, especially for predicting how species and ecosystems will respond to environmental change. However, identifying the extent of species’ distributions across terrestrial, marine, and freshwater habitats can be challenging because investigators may not fully understand which factors limit each species throughout their distribution. Distribution boundaries have been delineated using species distribution models based on occurrence information and/or habitat features, including mechanistic, process-based, and correlative models^1^.

**Figure 1:**
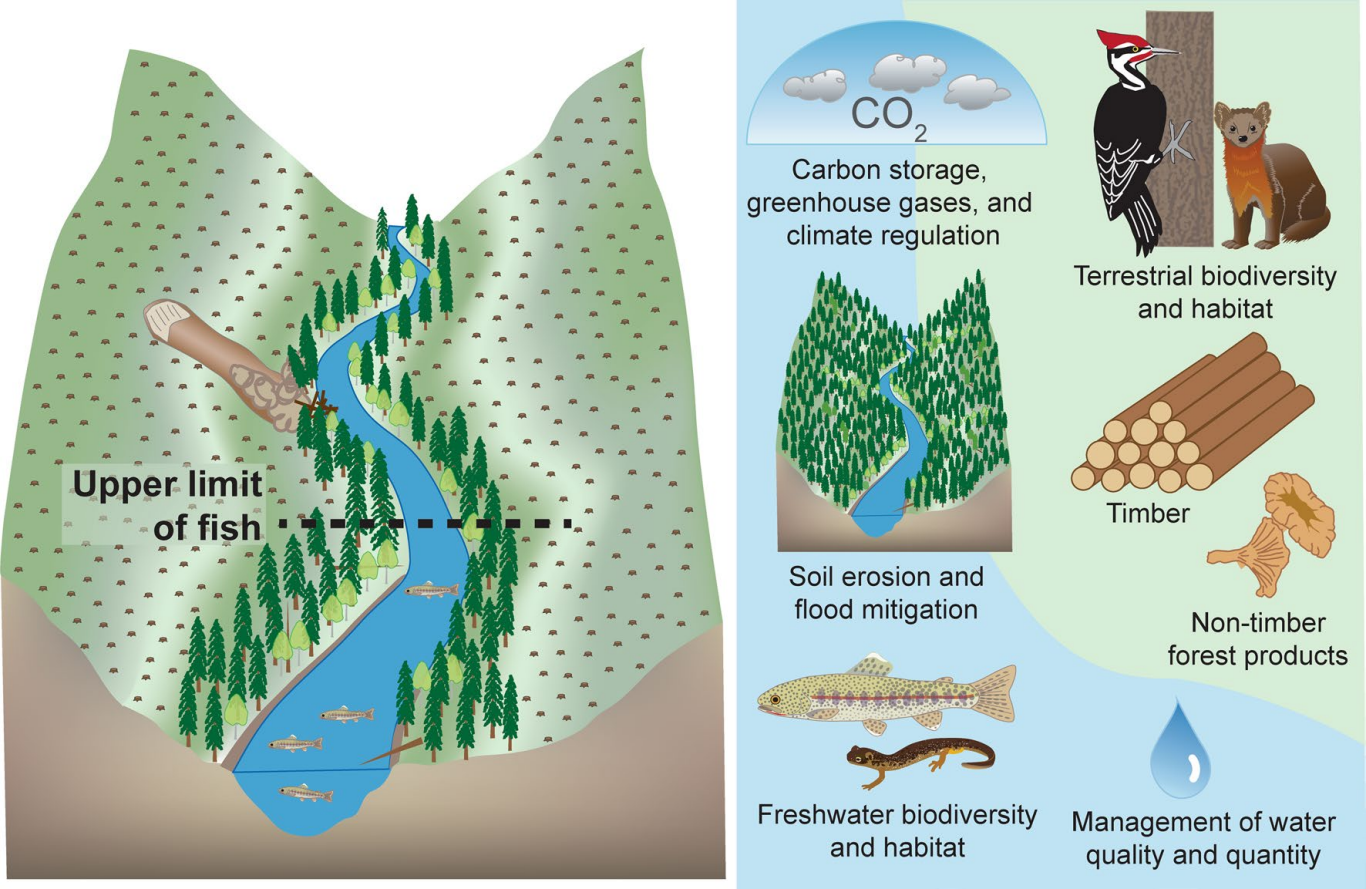
Ecosystem services at the upper limit of fish. Forest-freshwater ecosystems jointly produce benefits from nature, also known as ecosystem services, such as carbon storage, greenhouse gases, and climate regulation, aquatic and terrestrial biodiversity and habitat, mitigation of soil erosion and floods, timber and non-timber products, and management of water quality and quantity. Ecosystem services are jointly produced, thus when forests are harvested, the trajectory of ecosystem services associated with forest-freshwater ecosystems may change. Riparian buffer regulations for forest harvests depend on the upper limit of fish. Forest management practices near the upper extent of fish affect the levels of co-produced ecosystem services associated with the riparian buffer. [Figure developed in collaboration with coauthors by Kathryn Ronnenberg (USDA Forest Service, PNW Research Station)].

Land–water interactions highlight the complexities of understanding human impacts and human values associated with land-use, where forest harvest practices are regulated to protect important fisheries and streams. In western North America, ecosystem services provided by forests include fish and clean water, which are highly valued socially and economically. Balancing these sometimes-competing services has contributed to a rich evolution of research, regulation, and management^2, 3^. Consequently, an emphasis for contemporary forest and fisheries management practices is around the nexus of the upper distribution of fish (Fig. 1). For example, policies may impose costs in the form of forest harvest restrictions on lands adjacent to fish-bearing reaches owing to greater protections and wider riparian buffers than required on portions of streams without fish^4–6^. Regulations around forest harvest and their associated practices are designed to protect fish and their habitats while offering co-benefits to other species and to additional ecosystem services, such as water quality. Consequently, identifying the upper distribution of fish is ecologically, economically, and regulatorily relevant. A shared map that offers a visual context for where fish are, where they are not, and where their distributions end would help to navigate this tension and inform decision makers.

Fish species are distributed longitudinally within dendritic stream networks, and the upstream distribution boundary is driven by natural physical constraints. For example, the upper distribution of Coastal Cutthroat Trout (*Oncorhynchus clarkii clarkii*) is limited by a legacy of past anthropogenic activities including roads and culverts^7, 8^ in addition to stream size^9–11^, pool abundance^7^, channel slope^12–18^, and elevation^13^. High numbers of trout can be found in streams that are 1st- or 2nd-order^9^ with wetted channel widths of less than 6 m^10, 11^. Availability of pool habitats can extend the upstream distribution of trout higher in the stream network^7^; however, pools alone will not give an accurate indication of the uppermost presence of fish^12^. Channel slope or steepness can limit the upper extent of fish^12, 13^, with trout found in portions of streams with steeper slopes than co-occurring fishes^14^. Slopes of 20% are recommended as the cutoff for the uppermost fish across various western states and provinces of the U.S. and Canada, e.g.,^15–18^. Many geophysical and hydrological features are correlated with stream size, including elevation and streamflow, both of which can influence fish distributions. The upper distribution of fish in streams can also be influenced by conditions that affect streamflow permanence such as precipitation^13^.

Mapping the upper distribution limit of fish is complicated because differing land ownership, land use, and the methods and availability of survey data result in fish distribution maps that often reflect a mosaic of different methods at different scales. In North America, fish distribution maps are maintained by multiple entities, including private companies, states/provinces, and federal land managers, and are populated with different kinds of information depending on their mission and objectives. Fish occurrence documented by direct observation, often from electrofishing, trapping, or snorkeling, is the most definitive way to identify fish distributions. However, methods of direct observation can be labor intensive, rely on taxonomic expertise, are biased towards certain species and habitat conditions^19^, and they are influenced by both seasonal streamflow and the life cycle of the fishes, making sampling every stream reach across a region almost impossible. Consequently, consistency in data quality can also be an issue, especially across crews, agencies, protocols, streams, and regions. Some observational databases extend observed upper limits using expert opinion^11^, where potential fish distributions are extrapolated to upstream physical barriers that prevent fish movement. A newer technique of fish detections using environmental DNA could potentially facilitate occurrence documentation^10^ across broad regions. In addition, the usefulness of fish or habitat observations, regardless of source, depends on an adequate characterization of the stream network that provides a spatial context for the observations. For example, underestimating stream length has been shown to lead to underestimates in population sizes of an endangered trout (Apache Trout *O. apache*)^20^.

The upper reaches of the actual stream networks themselves are not consistently characterized across space because hydrography databases are not consistent if they are compiled from different sources that use varying methods to derive flowline hydrography^21, 22^. In mountainous landscapes, LiDAR-derived digital elevation models (DEMs) have been shown to better characterize the topographic landscape than traditional topographic maps with resolutions of 10 to 30 m or more. Using Light detection and ranging (LiDAR), the minimum spatial resolution of the DEM typically ranges from 0.5 m to 5 m. Consequentially, LiDAR maps more fully characterize the full extent of the stream network than DEMs based on topographic maps, advanced spaceborne thermal emission and reflection (ASTER), shuttle radar topography mission (SRTM), or photogrammetry. The existence of multiple distribution maps, multiple sources of information for fish observations, and multiple stream network databases presents a potentially confusing array of decision-support options for a land manager to work with, and may lead to different conclusions for management and conservation actions. This confusion could be minimized if a spatially explicit and consistently derived map of fish distributions existed on a standardized flowline hydrography that was both accurate and spatially contiguous across multiple land ownerships and with spatial scales ranging from the reach-levels used for operational planning up to regional scales used for strategic planning.

Modeling the upper extent of fish can broaden the spatial extent beyond that based on field observations of fish and address the planning needs of managers. Fransen et al.^13^ developed a two-stage trout distribution prediction model using logistic regression (LR) with hydro-topographic variables representing drainage area, elevation, and slope across private forest lands in western Washington. To streamline the modeling, they applied a stopping rule (SR) by identifying a discrete upper limit point for fish above which all stream reaches are classified as fishless to streamline the modeling. Although Fransen’s model^13^ is considered for predicting the upper extent of fish by managers and landowners, it was developed for a specific landscape, used a coarser stream hydrography of 10 m resolution (before LiDAR-derived hydrography became more prevalent in the region), and it has not been validated against fish observations for western Oregon.

**Figure 2:**
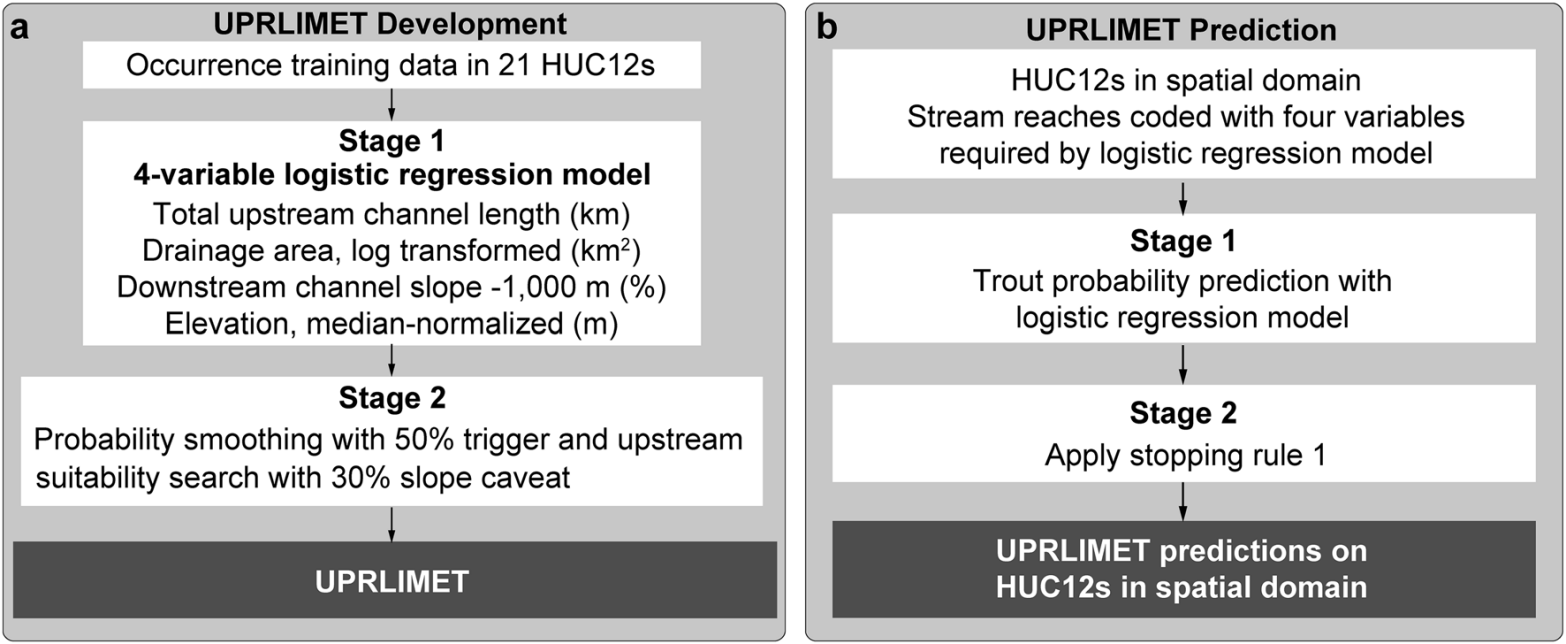
UPRLIMET (**a**) generalized development workflow and (**b**) prediction workflow. We constructed and compared 26 models to select the top performing model, termed UPRLIMET, based on the lowest overall error between observed and predicted upper extent of trout distributions across western Oregon. (**a**) Generalized development workflow for UPRLIMET, a single logistic regression model fit to trout occurrence observation data. Stage 1 involved fitting the 4-variable logistic regression to the occurrence observation data. Stage 2 included implementing Stopping Rule #1 (Fig. S1). (**b**) Generalized prediction workflow where the two-stage UPRLIMET prediction process is applied to all HUC12s in our study area producing a trout distribution map. The four environmental predictor variables in a and b are characterized at the scale of the individual reach (5–7 m) and derived from a 5-m LiDAR-derived digital elevation model (Data S1).

To address the need for a consistent approach to estimating uppermost distribution of fish in streams, we develop a spatially explicit Coastal Cutthroat Trout prediction model, UPstream Regional LiDAR Model for Extent of Trout (UPRLIMET; Fig. 2). Our primary objective is to improve predictions of the upper extent of fish in comparison to previous approaches, which were limited by computational power, data availability, and omission of headwater flowlines in the underlying hydrography. Here we present a novel model development and evaluation framework based on LiDAR hydrography that better accounts for headwater flowlines, examines permutations of 67 potential prediction variables representing hydro-topographic and climatic conditions in conjunction with three different modeling algorithms, and estimates predictive performance using nested spatial cross validation to account for spatial autocorrelation. Our secondary objectives are to assess the magnitude and impact of variable influences on upstream fish distribution limits and to compare these predictions across land ownership classifications. Using fish and upstream habitat observations collected from western Oregon, we build on detailed digital characterization of the geomorphic stream network and surrounding terrain from novel LiDAR data, we calibrate our model with observations from around 100 sites across federal, state, and private land ownerships throughout western Oregon, and demonstrate the utility of big data for identifying the optimal combination of 67 environmental predictor variables (Data S1) for predicting trout presence. We estimate predictive performance with a sophisticated Nested Spatial Cross Validation (NSpCV) routine, and compare mean absolute error (MAE), as estimated by the linear stream distance between predicted and observed upper-limit points, for 26 different approaches (Data S2; Data S3). We hypothesize that a prediction model based on habitat data and using a Random Forest (RF) model algorithm will provide superior predictive performance against observation data and other models, given the fixed nature of habitat information, many variables, and because random forest has demonstrated superior predictive performance in ecological applications^23–25^. By defining the factors that influence the upper limit of fish in the model, we identify biogeographic patterns, and inform decision makers about data gaps. This research supports strategies and policies for contemporary forest management and the conservation of freshwater fishes, especially in response to environmental change.

## Results

**Figure 3:**
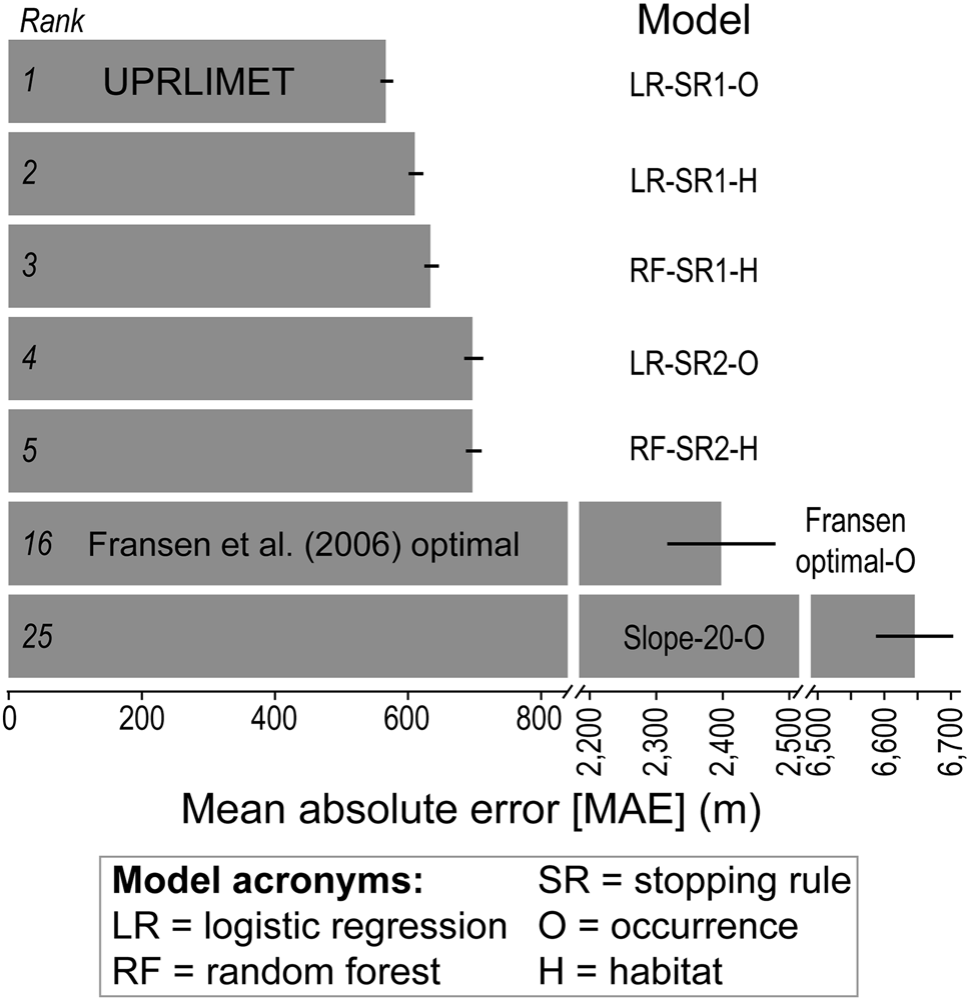
Comparison among selected models ranked by mean absolute error (MAE; m) of linear distance between the observed upper limit and the predicted upper limit. For the top five models, the model description specifies the development algorithm [*e.g.,* Random Forest (RF) or logistic regression (LR)], the stopping rule (SR) and its number (1, 2, or 3), and the type of training data [occurrence (O) or habitat (H)] used. In addition to showing the MAE for the top five models, two additional models are included, the Fransen et al.^13^ model, and a 20% slope cut off, where the lowest point on the network with a slope greater than or equal to 20% becomes the upper limit point. The model with the smallest MAE is called UPRLIMET.

The LR model based on the trout occurrence source information using stopping rule 1 (SR1; Fig. S1.; SI) was selected as the basis for UPRLIMET (Fig. 2) from a pool of 26 models (Data S2) formed from different algorithmic methods and containing different combinations of predictors, because it resulted in the lowest MAE (556 m), as measured by the linear stream distance between observed upper limit and predicted upper limit (Fig. 3, Data S3). An LR model based on habitat source information was ranked 2nd and a RF model based on habitat source information was ranked 3rd, suggesting that models with RF algorithms or based on habitat-source information are competitive. Computation time for the LR model was approximately 12 h less than for the 3rd ranked model that used RF. In comparison, upper limit estimates with the optimal Fransen model^13^ and a 20% slope cutoff produced MAEs of 2397 m and 6708 m, respectively, when compared to the occurrence observation data. Additionally the Refit (*i.e.* Refit-SR3-O; Data S2) of the optimal Fransen model produced an MAE of 2941 m when compared to the occurrence observation data, which was 544 m larger than the optimal Fransen model MAE (Data S3).

**Figure 4:**
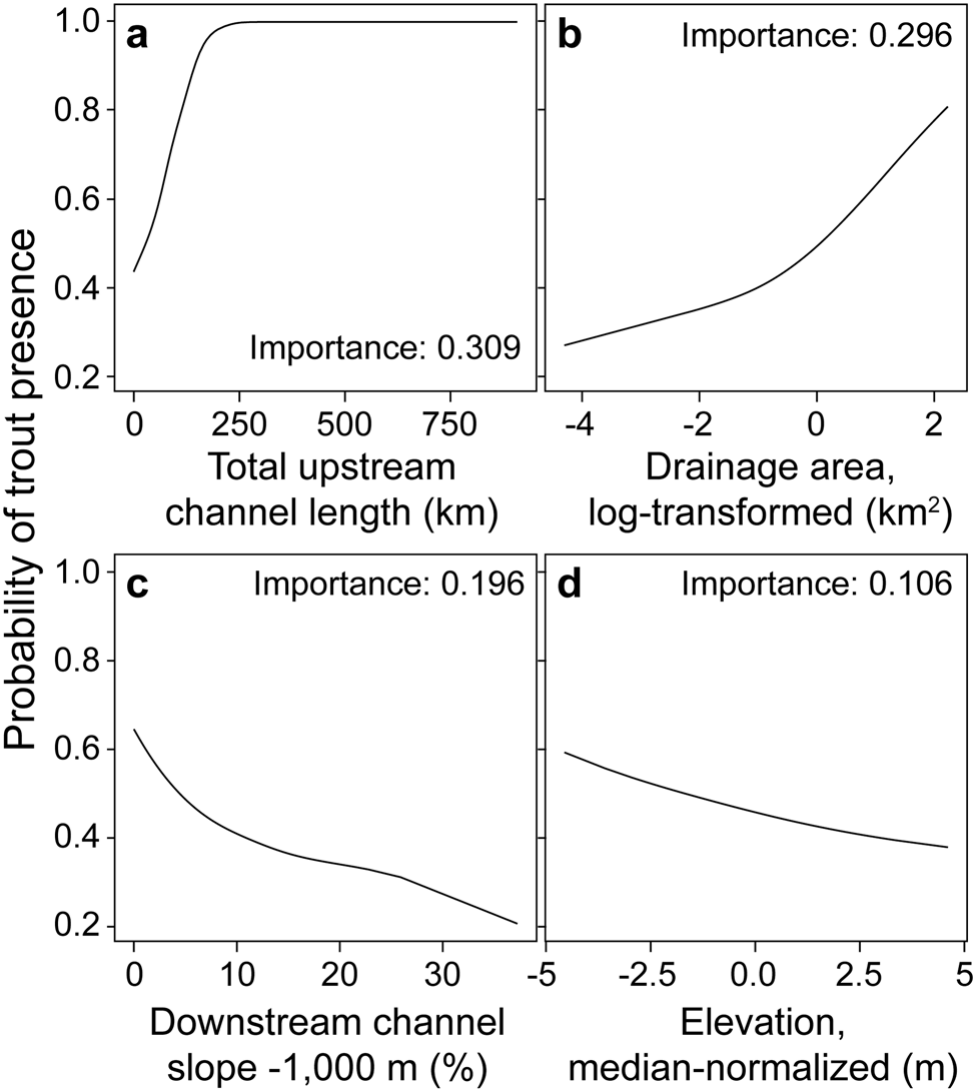
Partial-dependence profile plots of the four variables in UPRLIMET in relationship to the probability of trout presence, including (**a**) total upstream channel length (km), (**b**) drainage area, log transformed (km^2^), (**c**) downstream channel slope over 1000 m (%), and (**d**) elevation, median-normalized (m). Plots are arrayed in decreasing order of model importance.

UPRLIMET depended on four hydro-topographic predictor variables, presented in order of relative importance, to predict probability of suitability for trout presence: total upstream channel length (the sum of the stream length above the point of uppermost fish), drainage area (log-transformed), channel slope (downstream over 1000 m), and elevation (median-normalized; Fig. 4). For comparison, the optimal Fransen model^13^, which represents the state of the art in the region, also included drainage area, downstream slope, upstream slope, precipitation, and elevation. Between UPRLIMET and the optimal Fransen model^13^, drainage area was a key variable exhibiting a positive relationship with the probability of trout presence. Additionally, drainage area and downstream channel slope appeared to be important for predicting trout presence in general; it was key in both UPRLIMET and the optimal Fransen model^13^, and was important in many of the other models considered. Additionally, downstream channel slope was ranked as most important in two of the three feature-filtering algorithms and in the top three of all three algorithms. Drainage area ranked in the top three variables for two of the three filtering algorithms (Data S4).

We examined the relationship between these predictor variables and trout presence probability with partial dependence profiles. These profiles revealed that the probability of trout presence increased using increasing upstream stream length and increasing upstream drainage area (Fig. 4). When upstream length and drainage area were small, downstream slope as well as slopes greater than 9% became increasingly informative for characterizing reaches where trout were much less likely to be present.

**Figure 5:**
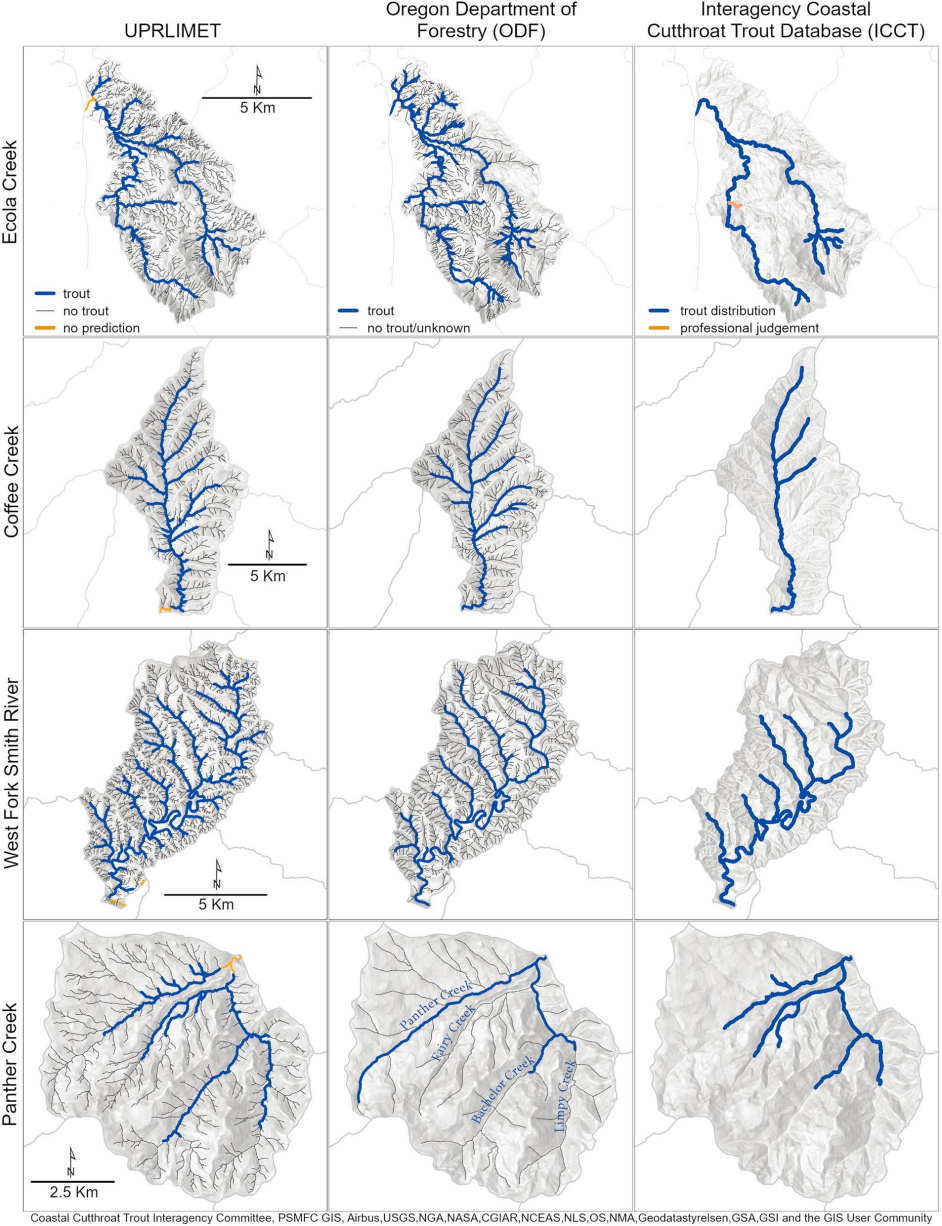
Examples of fish distributions in four HUC12 sub-watersheds, including Coffee Creek [Rogue River], Ecola Creek [Coast Range], and Panther Creek [North Umpqua River], and West Fork Smith River [Umpqua River]. Left panel shows predictions of presence and absence of trout using UPRLIMET. Middle panel shows trout occurrence and habitat distributions from Oregon Department of Forestry (ODF). Right panel shows trout occurrence distributions from the Interagency Coastal Cutthroat Trout (ICCT) database. The flowlines vary across the three databases owing to differences in hydrography associated with each database.

**Figure 6:**
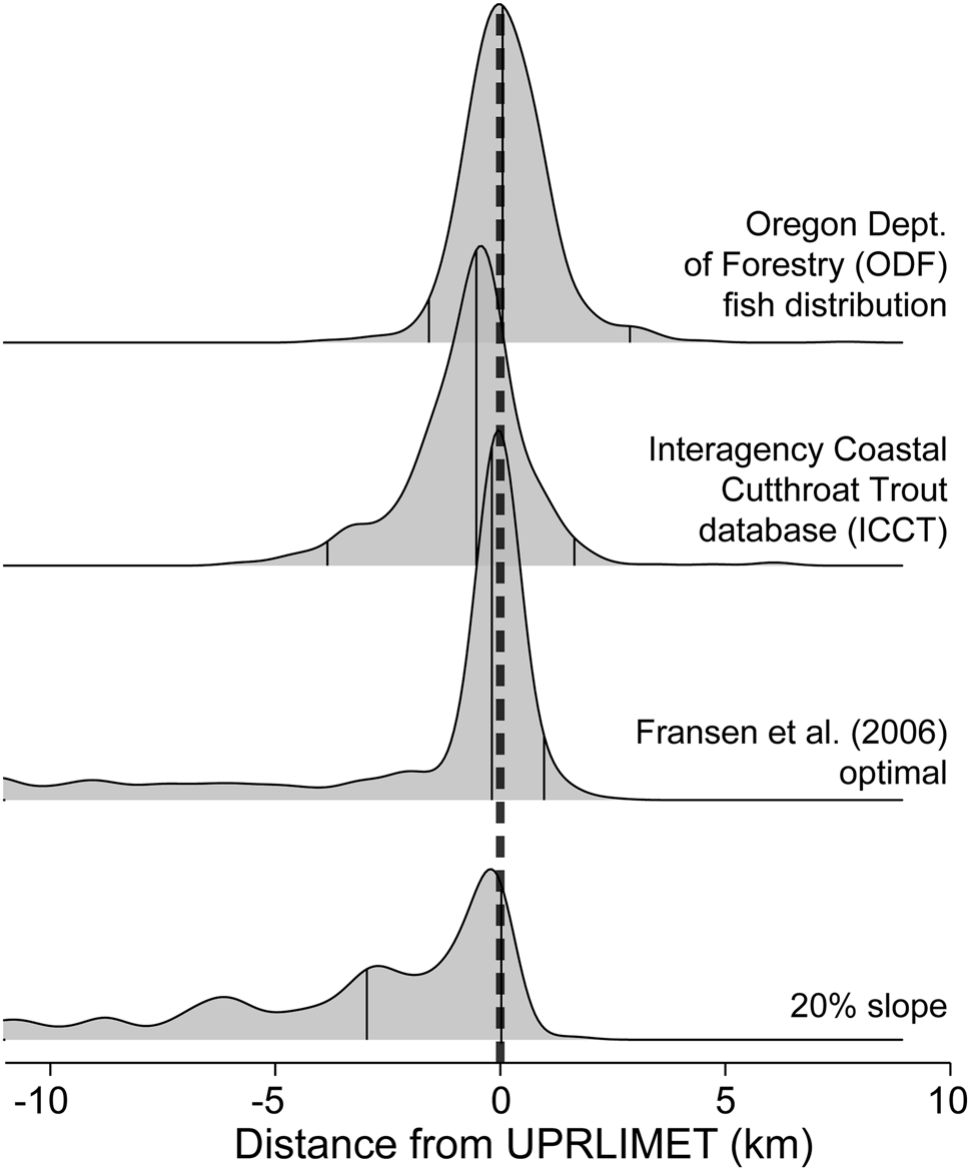
Ridge plots of frequency distributions of distances to the upper trout limit for UPRLIMET compared to trout occurrence and habitat distributions from Oregon Department of Forestry (ODF), trout occurrence distributions from the Interagency Coastal Cutthroat Trout (ICCT) database, Fransen et al. (2006) optimal model^13^, and 20% slope cutoff for western Oregon. Positive numbers represent overestimation relative to UPRLIMET and negative numbers are an underestimation. Note that the previously used estimates for upper trout limit by Fransen et al. (2006) optimal^13^, a 20% slope, and the ICCT database are biased towards underestimation and ODF overestimates. X-axis is distance to UPRLIMET in kilometers, and y-axis is relative frequency.

We compared distribution maps from UPRLIMET to the observations from the Oregon Department of Forestry (ODF) and Interagency Coastal Cutthroat Trout (ICCT). Although there was general agreement for overall distributions of trout, we found differences at the points of upper extent and for specific stream reaches (Fig. 5). ICCT is an occurrence-based dataset that was dependent upon fish ‘in hand’, whereas the ODF dataset used both occurrence and habitat information for identifying the upper limit of trout. For example, in the West Fork Smith River, the tips of the mid-stream terminal limits (Fig. S2) varied among distributions maps, however in the Panther Creek watershed both the tips of the mid-stream terminal limits and complete reaches were different. On Fairy Creek, the lateral limit was the upper limit for the ODF data, but not for the other two datasets, and on Bachelor and Limpy Creeks, the mid-stream terminal limits varied among the three distribution maps. A 20% slope cutoff and the optimal Fransen model^13^ under-predicted the upper limit of trout as did the Interagency Coastal Cutthroat Trout dataset, but with much less downstream error (Fig. 6). The ODF dataset over-predicted the upper limit of trout relative to UPRLIMET. In comparing upper fish distribution data in this manner, we noted some discrepancies that resulted from the differences in flowline hydrography underlying each dataset.

**Figure 7:**
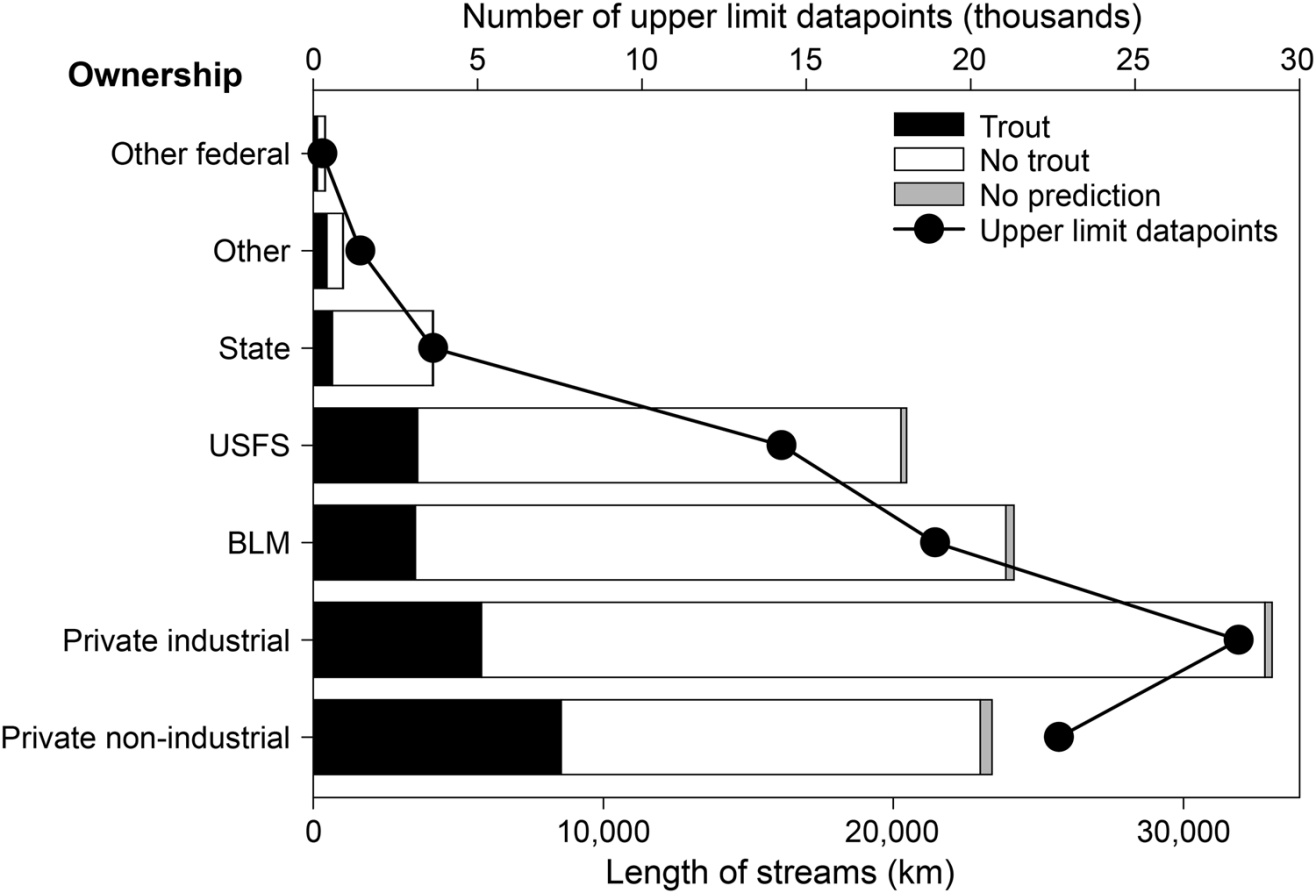
Distribution of the length of streams (km) with trout, with no trout, and with no predictions, along with the number of upper limit datapoints (thousands) predicted by UPRLIMET across land ownership categories of other federal, other, state, USFS (USDA Forest Service), BLM (Bureau of Land Management), private industrial, and private non-industrial. Stream length was estimated from the HUC12 scale. Note that streams without predictions occur when there is less than 1000 m of stream length over which to evaluate slope, or for channel-initiation reaches where upstream drainage area cannot be calculated.

Because UPRLIMET was continuous across the landscape, we were able to examine trends of upper limit of trout across land ownerships. UPRLIMET predictions revealed that private lands included more stream kilometers in general than other categories, and more fish-bearing streams than on state, Bureau of Land Management (BLM), USDA Forest Service (USFS), and other federal land categories (Fig. 7). Private industrial lands included the most distribution limit datapoints, whereas private non-industrial lands included the most fish-bearing streams and a disproportionately high percentage of upper-limit points relative to stream lengths.

## Discussion

UPRLIMET is our response to a need for a consistent method for predicting the upper extent of trout in all streams across land ownerships within our region. By developing and implementing the model using LiDAR-derived flowline hydrography, we offer a standardized, spatially explicit, spatially contiguous (where LiDAR hydrography is available), and high-quality fish-distribution layer based on the probability of fish presence. UPRLIMET maps both the probability of trout and the upper limit of trout across landscapes, ownerships, and jurisdictions, and better captures the upper extent of fish in headwater reaches relative to previous approaches allowing for a cross-boundary distribution map on which decision-makers and managers can base policies and regulations.

This work provides a transferable prediction modeling framework for systematically and comprehensively estimating the upper distribution limit of fish, which could be calibrated and implemented in watersheds and for fish species around the globe. Although the dependency on LiDAR-derived data here may be seen as a limitation to broader implementation of this method, the method is scalable to any resolution, and LiDAR is becoming increasingly ubiquitous in the United States through the U.S. Geological Survey 3D Elevation Program, which is funding LiDAR acquisitions across the United States. Furthermore, LiDAR data is available globally via data from GEDI and ICESAT-2 satellites that offer coarser resolution (~ 25-m) data that are still superior to either ASTER or SRTM derived-DEMs^26, 27^.

Minimizing prediction errors for the upper limit of trout is important to decision support and management planning because it ensures that forest-harvest regulations and management prescriptions are aligned. It is important to note that the prediction error estimates from this study are derived from the NSpCV process, except for models using 20% slope thresholds or unaltered parameterization of Fransen’s model^13^, because it is likely that the NSpCV estimates are conservative. They tended to overestimate error, as evidenced by the fact that the Refit model (*i.e.* Fransen’s optimal model^13^ refit to our data) exhibited a larger MAE than the unchanged optimal Fransen model^13^. This unexpected result was likely due to applying the NSpCV routine on the Refit model, resulting in the use of many intermediate models to characterize predictive performance using randomized subsets of independent training and test data. In contrast, the optimal Fransen model^13^ was developed independently using the data in this study and thus error could be evaluated directly without subsampling imposed by NSpCV.

The relatively low error for the two-stage model that becomes UPRLIMET suggests that it more accurately characterizes the upper limit of fish than all other models considered in this study, including the Fransen model^13^, which has been used for estimating upper limit of fish regionally. Although some of the models exhibited relatively small differences in error relative to the model that became UPRLIMET, small differences in predicted upper limit locations when considered in aggregate across multiple watersheds can potentially alter management decisions and expected outcomes. Differences in predictive performance and error between UPRLIMET and the optimal Fransen model^13^ are likely attributed to high-accuracy hydrography and hydro-topographic data (as LiDAR-derived DEMs were not available in western Washington in 2006), which allowed a finer-scale of analysis (*i.e.,* 5-m vs 10-m reaches). Additionally, the fact that UPRLIMET was fit to data solely from western Oregon likely offers predictive performance gains when applied to western Oregon when compared to the Fransen model^13^ that was fit to data from western Washington.

Quantifying the predicted accuracy associated with applying UPRLIMET to western Washington will require new data and is outside the intended scope of this study. However, we think it is reasonable to infer findings from UPRLIMET across regions with similar climatic and hydro-topographic conditions including northwestern California, western Oregon, western Washington, and southwestern British Columbia, especially given the broad availability of LiDAR-derived DEMs. This conclusion is supported the fact that both the Fransen^13^ and Refit models produced similar logistic regression coefficients (Data S5) and similar Matthews Correlation Coefficients (Data S6), suggesting that feature space of the two models is similar. This evidence is further corroborated by the high degree of overlap observed among the distributions of each of the four predictor variables for both western Oregon and Washington. We acknowledge that UPRLIMET does not contain identical predictor variables to Fransen’s model^13^ but maintain that they are similar enough in purpose that it is reasonable to assume that the feature space similarities are retained.

When we undertook this study, we hypothesized that a prediction model based on RF would offer superior predictive performance over those based on LR, given the availability of 67 predictor variables and RF’s demonstrated superior predictive performance in ecological applications^23–25^. However, our results suggests no improvement is offered by including more than four of the 67 environmental predictors examined, and that no clear advantage is offered by employing the more complex RF model, as evidenced by the top three of the top five prediction models being four-variable LR model algorithms (Fig. 3; Data S3.) The general importance of these variables to so many models is likely due to the strong linear relationships in the response of fish or no fish in logit space given the slopes of the curves in the partial dependence profiles (Fig. 4). This finding is congruent with the fundamental premise of LR, which is to explain and predict a response with a functional relationship, whereas RF deliberately focuses only on maximizing prediction accuracy with many decision trees^28^. Additional advantages to prediction models based on LR include the following: relatively better extrapolation performance over RF^29^, the simplicity of transferring a LR model to another processing platform using the model coefficients (versus the black box of RF decisions), and the immensely reduced computational processing times associated with LR model fitting and prediction. These advantages are especially key to this work, where there may be a desire to implement the model on other landscapes without the requisite expertise in doing so using the R software^30^. However, there are tradeoffs, as LR is more sensitive to the influence of outliers and multi-collinearity among variables, and overfitting is an increasing concern as the number of predictor variables increase, whereas RF tends to be robust to these concerns, but is more likely to produce a high-variance, low-bias prediction model^31^.

Although there is no single, general explanation for distribution limits of species^32^, the intersection of stream size, slope, and elevation together locate the upper limit of fish. Stream size corresponds to major ecosystem changes along a stream continuum including for energy sources, ecosystem metabolism, habitat characteristics, and biodiversity^33^, as well as the upper distribution limit of fish, as shown here. As expected, stream size accounts for the top two variables in the model suggesting that it is the major driver of the upper distribution limit of fish with the probability of trout increasing with increasing upstream stream length and upstream drainage area. Our finding proposes that downstream stream reaches are more likely to have fish. Although the underlying mechanisms have multiple influences, factors related to increasing stream size, such as increasing habitat size, habitat complexity, stability, or temperature variability^34^ have been shown to be important. Similarly, stream size is the most sensitive factor in intrinsic potential models for Chinook Salmon (*O. tshawytscha*^35^). Slope, the next variable of importance influencing the upper extent of fish, exerts control on physical habitats in streams, including channel morphology, hydraulics, sediment transport, substrate, and habitat^36^. Steep slopes drastically prevent trout from reaching areas above waterfalls or impassable chutes of over 25% slope, but trout can be found in streams channels without barriers at slopes as high as 28%^7, 14, 37^. Other fishes, such as Coho Salmon (*O. kisutch)* and steelhead (*O. mykiss)* are generally not found above 12% slope^38^. Interestingly, survival of fishes that make it upstream or are introduced above barriers may be facilitated by a geomorphic setting that is less prone to debris flows and other episodic sediment fluxes and has a greater resilience to flooding resulting from wider valley and greater floodplain connectivity^39^. Elevation or vertical topographic position may indirectly integrate broad influences of other landscape-scale or climate factors or also indirectly capture stream size, influencing the likelihood of fish presence. Frequently, species richness increases at lower elevations^40^, and we suggest that elevation also contributes to species distribution limits, as is the case for the Endangered Species Act listed Bull Trout (*Salvelinus confluentus*)^41^. The multiple factors associated with elevation correspond to the relationship found for stream size that smaller streams are less likely to have fish. Ultimately, the intersection of stream size, slope, and elevation guide us to finding the upper extent of fish in streams.

Physical influences have been proposed to be more limiting to fish distributions upstream, such as near the upper extent of fish, whereas biological factors are probably more important downstream^33^. Although 67 environmental predictor variables representing geologic, soil, climatic, and hydro-topographic conditions at local and patch scales are evaluated (Data S1), only the hydro-topographic variables of stream size, slope, and elevation are important to predicting the upper limit of fish in UPRLIMET. In fact, the top 9 models (Fig. 3; Data S3) relied on just four to five hydro-topographic variables, most of which were patch-scale variables or elevation at 1000 m, all of which incorporate a broader extent of influence. This suggests that local scale variables that contribute to fish limits, including slope or riparian influences may need to be further explored. In addition, some of the remaining 63 variables present in UPRLIMET, such as precipitation and air temperature, are important drivers of within-network trout distributions and contribute to their connectivity. Some of these predictor variables appear in the 10th ranked 26-variable RF-O-SR1 model (Data S2; Data S4; Data S8), but the influence appears to be dubious for isolating the upper limit and explaining variation in fish occurrence because MAE of upper limit was substantially higher than the 9 models with lower MAEs (Fig. 3; Data S3), and the lower MCC of the associated RF-O sub-model (Data S6). It is likely that other combinations of the 67 predictor variables, including precipitation, may be more important when this model development and evaluation framework is applied elsewhere, especially if those areas contain fishes or are places that are vulnerable to changing water temperatures and streamflow regimes. In addition, biological factors may be a concern in other watersheds, including invasive species and fish stocking which can limit the longitudinal distribution and the upstream extent of fishes.

Given the large geographic extent of this study, we expected other variables such as precipitation to be more important drivers, however due to a combination of a wet water year, a lack of precipitation gradient in the study area, coarse grain data, and location of fish in streams this was not the case. For example, 2017 was a wetter than normal water year^53^, and it may be that the gradient of precipitation variation in western Oregon was not strong enough to explain the variation in the spatial distribution of trout occurrence. All climate data, including the precipitation data were sourced from relatively coarse-scale (800 m) PRISM data. The inability to adequately downscale precipitation to characterize how precipitation truly varies within and between patches, especially along elevational gradients, likely confounded how the model interprets the influence of precipitation. Trout occurrence was on perennial streams, which is likely far enough downstream of locations where variation in precipitation was the dominant influence on streamflow permanence and consequently would not have been a factor.

Stream network structure plays a key role in the upper limits of fish. Upper limits for fish can occur at either lateral or terminal points^13^ and when mapping these points, differences were seen for UPRLIMET relative to other datasets. Lateral limits end in the tributary stream just above where it connects with a mainstem stream. Terminal limits include both mid-stream terminal limits where fish drop out in the middle of a stream channel owing to a soft (i.e., transient barrier or puttering out) or hard (i.e., waterfall) edge, and confluence terminal limits where the upper limit of fish ends at the confluence. For example, when closely examining the 14 watersheds where we have overlapping information across various datasets and models, UPRLIMET and the Fransen optimal model^13^ exhibit substantial agreement in their lateral limits. However, the largest differences are in their terminal ends, especially terminal mid-stream limits, probably owing to hydro-topographic changes that contribute to fish occurrence at confluences, which are more pronounced than mid-stream. Accordingly, the logic in the stopping rule is likely important in identifying specific upper extent of fish distributions in reaches that end mid-stream.

Differences among databases for the upper distribution limits of fish come from both the upper limit points and depiction of fish-bearing reaches, underscoring the importance of having a shared map with common coverage of the fish extent across landscapes and ownerships. Differences among mapped distributions can result from source information, relating to whether it is modeled or occurrence data. Models, such as UPRLIMET, can be applied across a broad extent based on model parameters and training data, thereby offering broad coverage for distributions (and quantifiable error) across the landscape, ownerships, and jurisdictions. However, models are limited by accuracy and fit. As such, they can incorrectly predict distributions in some areas, especially if there are prediction features not yet trained with the model data where prediction would require extrapolation of the model. This makes both the training dataset and modeled extent important considerations, as models are only as good as the data used to develop them. Updating UPRLIMET with new data as it becomes available will help to expand the prediction domain, improve accuracy, and allow the model to do more interpolation than extrapolation.

Distributions based on occurrence information depend heavily on data availability, data quality, and access. Differences in data availability can lead to inconsistent coverage across landscapes and ownerships, with high coverage in some watersheds and low to no coverage in others. Inconsistent coverage can lead to errors that are difficult to quantify across landscapes, ownerships, and survey crews. Occurrence information also depends on the ability to survey watersheds and gain access across ownership types, including on private lands that do not have the same assurances of access as public lands, resulting in information asymmetry^42, 43^. Data quality also depends on the spatial accuracy of the points of uppermost fish, which are a function of GPS quality and error, and can drastically change the modeled results, as these points are used in the training dataset. Differences among mapped distribution limits also result from differences in field protocols on designating last fish. For example, some crews note fish distribution limits where they visually see the last fish, whereas others note it upstream of where they saw last fish, based on habitat features that would limit fish. With the advent of LiDAR-derived DEMs and associated LiDAR-derived stream hydrography, like those available in much of western Oregon, have revealed additional flowlines in watersheds compared to previous topographic maps, which adds more potential tributaries to survey for fish-distribution assessments. When these new previously unmapped tributaries are paired with a model, such as UPRLIMET, a common information set is available across landowners, managers, and agencies for the upper extent of fish. This helps policymakers determine where to apply regulations that support fisheries and forest management, based on the upper fish limit.

Next steps for applying and expanding the model include addressing current data gaps. More information and observations about the upper distribution limits of fish beyond western Oregon would be needed to properly expand the spatial scope of the model. The upper extent of fish is at the detection limit of many current technologies, including global nativation satellite system (GNSS), geographic information systems (GIS), and LiDAR, especially in forested landscapes. Better precision of GNSS coordinates from observations would help greatly. From an ecological perspective, we could focus on fish distribution limits that vary seasonally or interannually to better understand which stream features and hydrologic parameters influence those endpoints. We also need information related to locations of barriers, including culverts, waterfalls, and knickpoints to understand their influence on contemporary distributions. Incorporating variables representing riparian conditions as well as leveraging higher-resolution DEMs (< 1 m) to better capture fine-scale geomorphic conditions such as pools and small barriers, especially in the headwater reaches, has the potential to further enhance the ability to resolve upper fish limits.

There are also opportunities to refine the underlying modeling methodology. Deep learning methods applied to structured data (e.g., data tables like those used in this study) are showing significant improvement over RF and gradient boosting methods^44^, which may result in improved upper limit estimations because of the potentially improved prediction of trout distributions. Given that observation data is typically collected in advance of localized management operations, there may be advantages to implementing a Bayesian Updating approach that could readily utilize new data, versus having to re-fit models each time new data become available^45^. A more in-depth analysis of different variable combination and model development algorithms might be possible via implementation of bias correction bootstrapping cross validation routines, which are considerably less computationally intense than our NSpCV routine^46^. However, given the significant changes in error imposed by simply applying different stopping rules (Data S3), and the fact that most of the classification algorithms were producing greater than 90% prediction accuracy, it seems likely that refining the post-processing upper limit method by applying a secondary classification model, local maxima search algorithms, or additional conditional logic routines, may yield the greatest net reduction in error with a relatively low development input.

In conclusion, we offer a prediction model development and evaluation framework for how to systematically consider the upper distribution limit of fish that could be broadly applied to watersheds and fishes. Distribution boundaries are fundamental for species conservation as well as for understanding how species might respond to environmental change; policymakers and managers reference distribution maps to determine management decisions, policies, and regulations. Distribution of fish influences policies can impose costs in the form of forest harvest restrictions and benefits in the form of ecosystem services, including co-benefits to other species found with fish, including crayfishes, stream-living amphibians, and mussels. UPRLIMET offers modeled trout distributions across the landscape and land ownerships through a shared map, stream flowlines, and data sources, all of which can be updated as new data is gathered. With this comprehensive prediction model development and evaluation framework, we (a) improve the information available to policymakers and managers by incorporating the best available LiDAR-derived hydro-topographic data, (b) train the model using field observations, (c) compare our findings with other methods that managers are using for estimating fish distributions (e.g. Fransen et al. (2006)^13^ and 20% slope threshold), and (d) contrast UPRLIMET prediction results with multiple fish distribution datasets being used to identify the upper extent of fish. The availability and use of common models, data, and maps across land ownerships will streamline policy and management planning and activities.

## Methods

### Study area

The fish species found in the uppermost segments of streams across the Pacific coast of North America are commonly Coastal Cutthroat Trout^11, 13, 14, 48^, although in a few streams, *Cottus* spp., juvenile steelhead *O. mykiss*, or juvenile Coho Salmon *O. kisutch* can be the uppermost fish. Consequently, Coastal Cutthroat Trout are identified as the focus of monitoring for fish taxa at the upper extent and they are the species we consider here.

In 2017, we sampled populations of Coastal Cutthroat Trout that were randomly selected in historical surveys in 1999 and 2000^49, 50^ from across western Oregon’s ecoregions (including physiographic provinces and geologies) and were found above barriers. By sampling these trout populations, we were informed as to where to start the current assessment allowing us to assess the uppermost fish occurrence and identify habitat barriers on populations across western Oregon on private, state, and federal lands. In spring and early summer (5 April–29 June 2017), we visited 103 streams where we collected field observations that consisted of coordinates of uppermost fish occurrence and nearest upstream habitat barrier using a consumergrade^45^ GNSS receiver. We initiated surveys at 175 m downstream from the uppermost fish coordinates noted in the 1999 and 2000 surveys^49, 50^ to account for the possibility that uppermost fish could be downstream of the earlier surveys. We used a spatially continuous, single-pass backpack electrofishing approach^49, 52^ as it is the method protocol typically used at the upper extent of fish and has been shown to be equally effective at sampling in this context. We sampled across all available habitats similar to other studies^11, 12^. The uppermost fish occurrences with electrofishing were made by visually identifying individual fish to species, which is representative of surveys generally used to determine the occurrence of the uppermost fish in streams. When the uppermost fish was detected, we continued to sample for at least an additional 45 m upstream and 6 pool habitats^18^. Habitat data included the location and type of habitat barrier observed at or upstream of the uppermost location with fish detection.

Owing to the complex and dendritic nature of the stream network in mountainous western Oregon, we wanted to ensure that the DEMs and stream hydrography used for this work were the best available representations of the hydrologic landscape. As such, the spatial domain for this study was limited to the 383 USGS 12-digit hydrological unit code (HUC12) sub-watersheds in western Oregon that had LiDAR-derived DEMs and associated LiDAR-derived hydrography in the National Hydrography Dataset (NHD)^22^. HUC12s were chosen because this is the smallest unit in which NHD updates are published in Oregon^54^. Because LiDAR-derived streams were not available for some study sites, three sites were dropped for model development, resulting in 100 occurrence (O) and 99 habitat (H) observations across 21 HUC12 sub-watersheds (Fig. S3).

Thirty-year average precipitation by water year for our study area ranged from 119.4 cm in the Rogue-Sisk iyou basin to 227.1 cm in the Coast Range. Total precipitation during the 2017 water year, when new observations were collected, was higher than normal, ranging from 148.6 cm in the Umpqua River basin to 299.2 cm in the Coast Range^53^.

### Alignment of field observations to LiDAR-derived hydrography

All field observation data were aligned to NHD flowlines (*i.e.,* streams) to facilitate model development. Alignment was necessary because some of the usable observations were as much as 30 m from the nearest NHD flowline. The alignment process involved a review in ArcGIS Pro GIS software with the assistance of the technician who collected the observation data. All data points were overlaid on top of a LiDAR-derived hillshade^55^ and individually examined to determine appropriate placement within the stream network. As of September 2021, the U.S. Geological Survey (USGS) National Hydrologic Dataset (NHD)^22^ is the publicly available centralized database for these LiDAR-derived hydrography, and it currently only covers about 30% of the landscape west of the Cascade Crest^21, 23^.

### Development and prediction of UPRLIMET

The UPRLIMET development and prediction process is described in the paragraphs below, with three of the sections describing the development process and the fourth describing the prediction process. The development process is constructed with the aim of addressing 10 questions (Data S7) related to understanding applicability of the Fransen model^13^ to western Oregon and identifying opportunities to improve predictive accuracy with other combinations of predictor variables and model development algorithms.

1. For each of the 21 HUC12s with training observations, we compiled spatial data of 67 potential environmental prediction variables (Data S1) thought to characterize different factors that influence the upper limit of trout, especially those related to stream flow permanence and barriers to fish passage^13, 23^. Variables included 5 m resolution hydro-topographic (e.g. channel slope, drainage area, surfacer roughness, etc.) derived from LiDAR DEMs^55^, and 800 m resolution climate (e.g. precipitation, air temperature) data for the 2017 calendar year as well as the 30-year climate normal period^56, 57^. A major constraint on data inclusion was that it was available continuously across the entire spatial extent of the study area and hydrography, which had the effect of limiting incorporation of stream temperature from NorWest^58^ as well as other potentially important biotic and abiotic drivers such as competition and connectivity. Data were characterized at the local (at the point or reach) and patch scale, where the patch refers to the drainage basin upstream of any given reach. Patch-scale variables were parameterized flow conditioned parameter grids (FCPGs)^59^ which represent the upstream drainage area average of the variable value along each 5 m grid cell along the stream. LiDAR-derived NHD flowlines representing the stream network were then aligned to a 5-m resolution DEM that was the source of hydro-topographic data and split the network into 5- to 7-m reaches (Data S1) to ensure spatial agreement with the environmental prediction variables. Reach length varied depending how a given flowline crosses the corresponding DEM grid cells (7 m along diagonals). The 67 predictor variables were indexed to individual reaches. Reaches were then coded with a presence response variable containing binomial “trout” or “no trout” classifications propagated from upper-limit observation data for both O and H observation data types. Data were then balanced by undersampling the majority class to ensure equal numbers of “trout” and “no trout” reaches in each HUC12. Balancing ensured that the resulting trout presence prediction sub-models described in (2) below were developed such that the decision boundary between “no trout” and “trout” on the resulting 0% to 100% presence probability distribution was centered at 50%.
2. We developed eight trout presence prediction sub-models (Data S2) composed of four sub-models for each observation data type (i.e. O and H), and evaluated predictive performance in terms of Matthews Correlation Coefficient (MCC) using a nested spatial cross-validation (NSpCV) routine (SI); Data S2). The four basic sub-models were the Fransen optimal model^13^, a refit of that model to the observation data presented here, an optimal Random Forest (RF) model based on the combination of 67 predictor variables resulting in the highest MCC, and an optimal Logistic Regression (LR) using the combination of 67 predictor variables resulted in the highest MCC^60^. MCC was used here because it has been shown to be robust to the many of the conditions that can confound interpretation of predictive performance^61^. The upper limit as determined by O observations tended to be spatially distinct from the upper limit determined by H observations, resulting in the need to develop distinct sub-models for each training data type (SI). Sub-models were designed to test specific questions concerning upper limit of trout including, but not limited to examining whether increasing data dimensionality (e.g., 67 predictor variables and LiDAR-derived flowlines) produced a more accurate prediction model than the one specified in Fransen et al. (2006)^13^ (Data S6). LR and RF model development algorithms were chosen because of their previous use in related stream modeling applications^13, 23^. LR is a form of a generalized linear model (GLM) development algorithm that fits a log-linear relationship between variables and the binomial training data using a logit function to convert the binary categorical response of “trout”, “no trout” into a continuous distribution from 0 to 1. For this, we used the Glmnet implementation in R^62^. RF^63^ is a machine-learning model-development algorithm that uses an ensemble of weakly correlated decision trees to ascribe a response classification to a combination of variables. It tends to be computationally more intense than LR, but robust to many of the distributional assumptions that can affect LR outcomes.
  The NSpCV routine was a complex process that used a 5-repeat fivefold spatial resampling approach to produce estimations of sub-model MCC that are robust to the over optimism associated using conventional non-spatial approaches on spatially correlated data^64^ (SI). O and H training data were each grouped into spatial blocks by HUC12 under the assumption that data within a HUC12 were more correlated than data among HUC12s, in terms of the relationship between the predictor variables and the response (i.e., “trout” or “no trout”). For a given sub-model, each of the five repeats were randomly assigned spatial blocks of training data to one of five folds, where folds became training and validation subsets. An intermediate sub-model was fitted to data in four of the five folds, then validated against the held-out fold to produce an estimate of MCC when whole HUC12s were excluded from the model. The fit and validation process was repeated until all five folds were evaluated. All RF sub-models included a hyperparameter tuning routine nested within each fivefold resampling routine to estimate unbiased estimate of predictive performance^65^. The NSpCV routine produced 25 intermediate sub-models, each paired with an estimate of MCC, and a validation data subset. Intermediate sub-models were critical to understanding predictive performance in HUC12s not constrained by the model and were key for the error assessment in Sect. (3) below.
3. We predicted upper limit locations for each of the 21 HUC12s in the training domain with 26 models (13 for each of H and O data types; Data S2) and estimated Mean Absolute Error (MAE; Eq. 1) between the observed upper limit location and the predicted upper limit location by calculating linear stream distance between the two locations. Twenty-four of the 26 models were two-stage models resulting from combining each of the eight trout presence prediction sub-models in (2) above, with each of three stopping rules (SRs; SI). The purpose of SRs, as described in Fransen et al.^13^, was to classify stream reaches into binomial “trout”, ‘no trout’ classes based on the predicted trout presence probability at each reach while accounting for upstream and downstream conditions such that only a single point on a stream is the upper limit of fish. SR1 was a variation on Fransen’s optimal stopping rule^13^, with the major changes being the application of a rolling average to smooth the predicted probabilities and, because the training data were balanced, setting of the cut point (i.e., probability threshold) for identifying upper limit at 50% (SI Step; Fig. S1). SR2 used the lowest point on the stream having a probability ≥ 50%, to identify the point of upper limit, which is analogous to Fransen’s benchmark 1 stopping rule^13^. SR3 is the optimal stopping rule described in Fransen et al.^13^. The remaining 2 of the 26 models were identical to each other, but resulted in different MAE estimates because they were compared separately to either the O or H data. These two models apply a rule used in Oregon when observation data were not available^18^ that defined the upper limit of trout as the lowest point on a stream just downstream of a 20 m run of stream having a slope greater than or equal to 20%.
  1 $${MAE }_{{s}_{r}}= \frac{\sum_{i=1}^{{{n}_{s}}_{r}}|{y}_{{i}_{{s}_{r}}}-{x}_{{i}_{{s}_{r}}}|}{{n}_{{s}_{r}}}$$
4. We selected the UPRLIMET model from the 26 models described above using lowest MAE as the criterion. We then generated predictor variable data for all 383 HUC12s in the prediction domain and applied two-stage UPRLIMET model to predict upper limit of trout for each stream reach (Data S2; Fig. S4) in each HUC12 in the prediction domain.

Equation 1: Mean absolute error [MAE]—Where *i* is an observed upper limit from *n* upper limit observations in subset *s of* the training data where *s* corresponds with each of 5-folds within each *r* of 5 repeats of the Nested Spatial Cross Validation [NSpCV] routine. *y* is the linear stream distance (m) of the observed upper limit point from the HUC12 outlet for a given model, and *x* is the linear stream distance (m) of the predicted upper limit point and the difference between *y* and *x* represents error in units of meters (m).

### Evaluating UPRLIMET

To provide an ecological context to UPRLIMET predictions, we used the DALEX package in R^65^ to generate partial-dependence profiles and variable importance scores, which depicted how probability of trout presence changes as a function of the predictor variables, and the relative importance of each variable for predicting trout presence, respectively.

We compared upper-limit predictions to four other sources of fish-distribution data in 14 randomly selected watersheds within our study area. This allowed us to provide a management-relevant understanding of UPRLIMET performance against current data. These sources were: (1) the ICCT dataset^67^; (2) the ODF fish layer; (3) predictions calculated using the optimal Fransen model^13^; and (4) predictions of the upper limit based on the downstream-most presence of a 20% or greater slope over a 20-m run of stream. For these analyses, we calculated the linear stream distance between the UPRLIMET predicted upper limit and the points from the other three upper-limit data such that negative values correspond with points that fall downstream, and positive, points upstream of UPRLIMET predictions, respectively. We mapped comparisons of trout distribution and upper-limit locations for four of these HUC12s.

Finally, we conducted an analysis of UPRLIMET predictions on the HUC12s in our prediction domain by land ownership (*i.e.,* private industrial, private non-industrial, state, US Bureau of Land Management, USDA Forest Service, and other federal) to provide additional social context and identify trends that may be useful for planning and management purposes. For this analysis, we summarized both predicted trout distributions (presence and absence in terms of total length by ownership, and distributions of predicted upper-limit points in terms of frequency of occurrence by ownership.

## Supplementary Information

Supplementary Information 1.

Supplementary Information 2.

Supplementary Information 3.

Supplementary Information 4.

Supplementary Information 5.

Supplementary Information 6.

Supplementary Information 7.

Supplementary Information 8.

Supplementary Information 9.

Supplementary Information 10.

## Data Availability

The datasets analyzed during the current study are available in the Forest Service Research Data Archive, https://doi.org/10.2737/RDS-2022-0087.
