# Author Correction: UPRLIMET: UPstream Regional LiDAR Model for Extent of Trout in stream networks

**DOI:** 10.1038/s41598-023-31164-z

**Published:** 2023-03-10

**Authors:** Brooke E. Penaluna, Jonathan D. Burnett, Kelly Christiansen, Ivan Arismendi, Sherri L. Johnson, Kitty Griswold, Brett Holycross, Sonja H. Kolstoe

**Affiliations:** 1 U.S. Department of Agriculture, Forest Service, Pacific Northwest Research Station, 3200 SW Jefferson Way, Corvallis, OR 97331 USA; 2 Department of Fisheries, Wildlife, and Conservation Sciences, Oregon State University, 104 Nash Hall, Corvallis, OR 97331 USA; 3 Department of Biological Sciences, Idaho State University, 921 S. 8th Ave Mail, Stop 8007, Pocatello, ID 83209-8007 USA; 4 Pacific States Marine Fisheries Commission, 205 SE Spokane St., Portland, OR 97202 USA; 5 U.S. Department of Agriculture, Forest Service, Pacific Northwest Research Station, 1220 SW 3rd Avenue, Suite 1410, Portland, OR 97204 USA

Correction to: *Scientific Reports* 10.1038/s41598-022-23754-0, published online 01 December 2022

The Supplementary Information 1 file published with this Article contained an error, where three additional sentences at the end of the ‘Step 3 – *Stopping* Rule Development’ section were omitted. The original Supplementary Information 1 file is provided below.

This error has now been corrected in the Supplementary Information 1 file that accompanies the original Article.

## Supplementary Information

Supplementary Information 1.

