# Supplementary material for: UPRLIMET: UPstream Regional LiDAR Model for Extent of Trout in stream networks
Source: Sci Rep. 2022 Dec 1;12:20266. doi: 10.1038/s41598-022-23754-0 (PMC9715699; doi:10.1038/s41598-022-23754-0)
Supplement: Supplementary file 1 — Supplementary Information 1. [file 41598_2022_23754_MOESM1_ESM.docx]

Supplementary Information

**UPRLIMET: UPstream Regional LiDAR Model for Extent of Trout in stream networks**

Brooke E. Penaluna^1^*, Jonathan D. Burnett^1^, Kelly Christiansen^1^, Ivan Arismendi^2^, Sherri L. Johnson^1^, Kitty Griswold^3^, Brett Holycross^4^, and Sonja H. Kolstoe^5^

^1^U.S. Department of Agriculture, Forest Service, Pacific Northwest Research Station, 3200 SW Jefferson Way, Corvallis, OR 97331, USA

^2^Oregon State University, Department of Fisheries, Wildlife, and Conservation Sciences, 104 Nash Hall, Corvallis, OR 97331, USA

^3^Department of Biological Sciences, 921 S. 8th Ave Mail, Stop 8007 | Pocatello, ID 83209-8007

^4^Pacific States Marine Fisheries Commission, 205 SE Spokane St., Portland, OR 97206

^5^U.S. Department of Agriculture, Forest Service, Pacific Northwest Research Station, 620 SW Main Street, Portland, OR 97205, USA

*Author for correspondence, email: [brooke.penaluna@usda.gov](mailto:brooke.penaluna@usda.gov)

**Supplementary Figure S1**

**Supplementary Figure S2**

**Supplementary Figure S3**

**Supplementary Figure S4**

**Supplementary Data S1**

**Supplementary Data S2**

**Supplementary Data S3**

**Supplementary Data S4**

**Supplementary Data S5**

**Supplementary Data S6**

**Supplementary Data S7**

**Supplementary Data S8**

**Supplementary Methods**

**Supplementary Literature Cited**


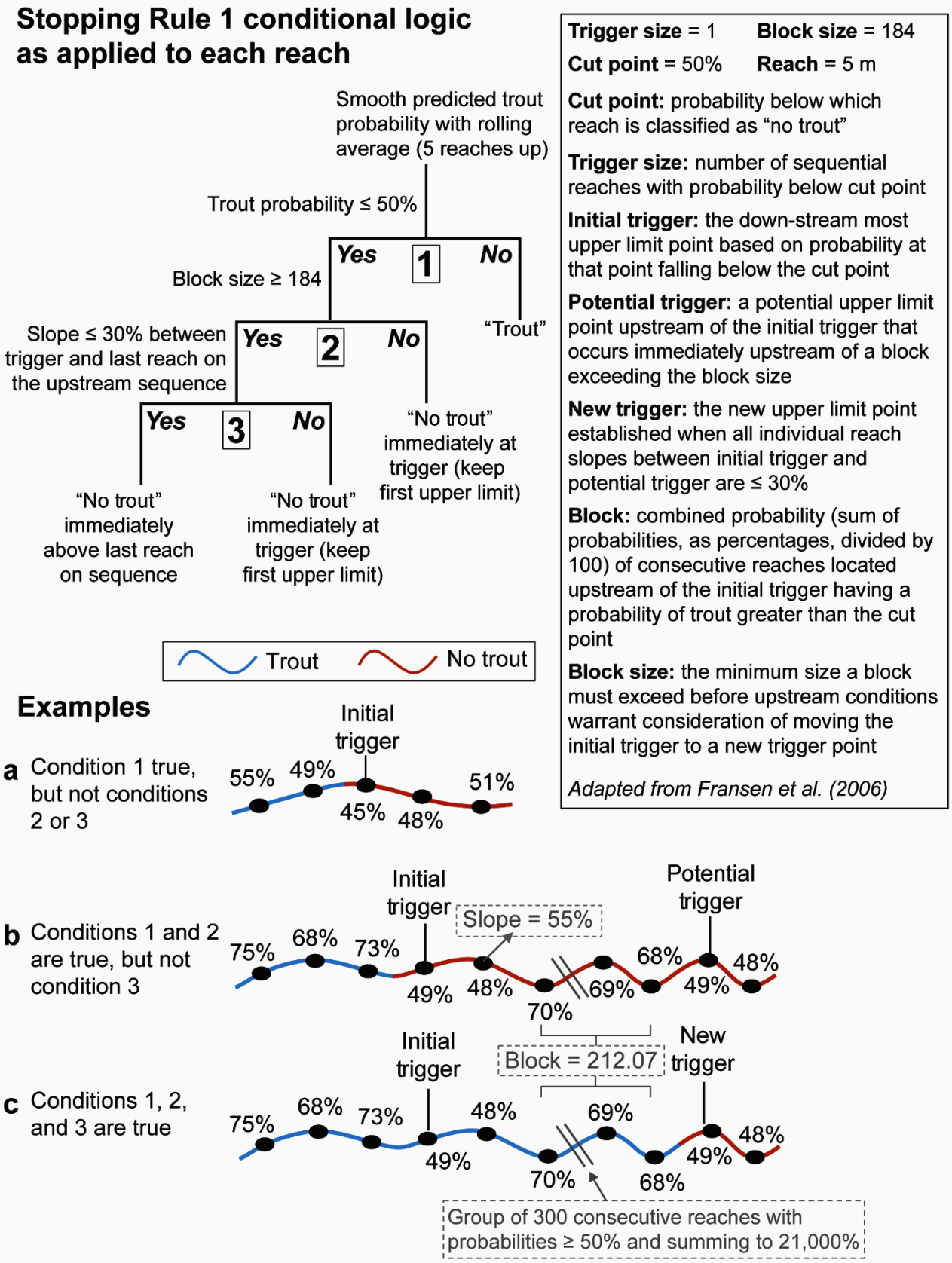


Figure S1. Stopping rule 1 logic. Graphical depiction of the logic applied to each stream reach in UPRLIMET to make a determination of ‘Trout’ or ‘No Trout’. Examples are paired with each of the conditional logic statements to depict how the upper limit point and stream reach classification changes depend on the logic.


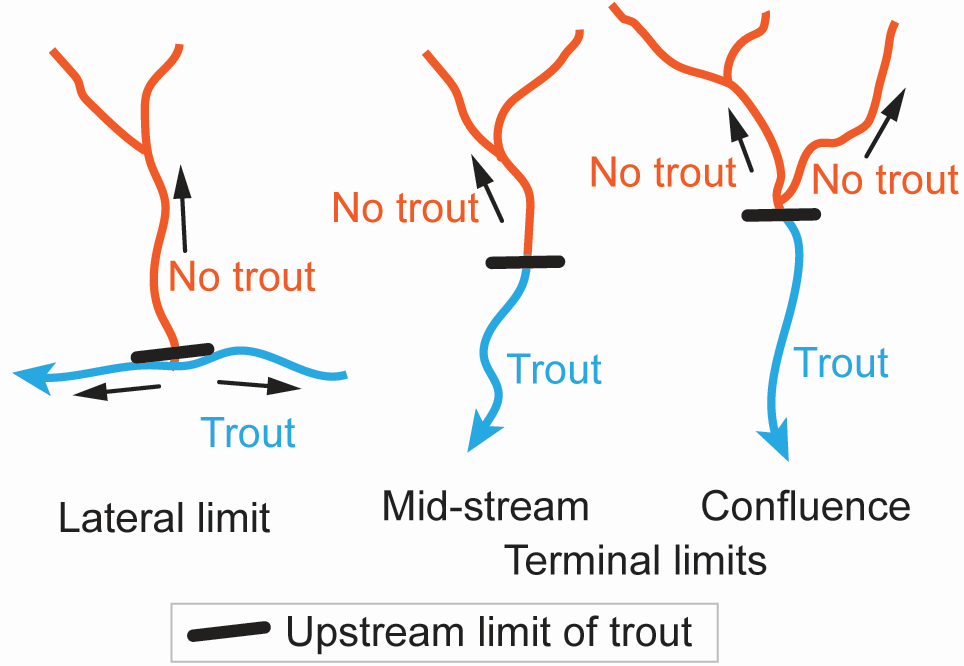


Figure S2. Lateral and terminal upper limits of fish. An illustrative example showing lateral upper limits, mid-stream terminal limits, and confluence terminal limits.


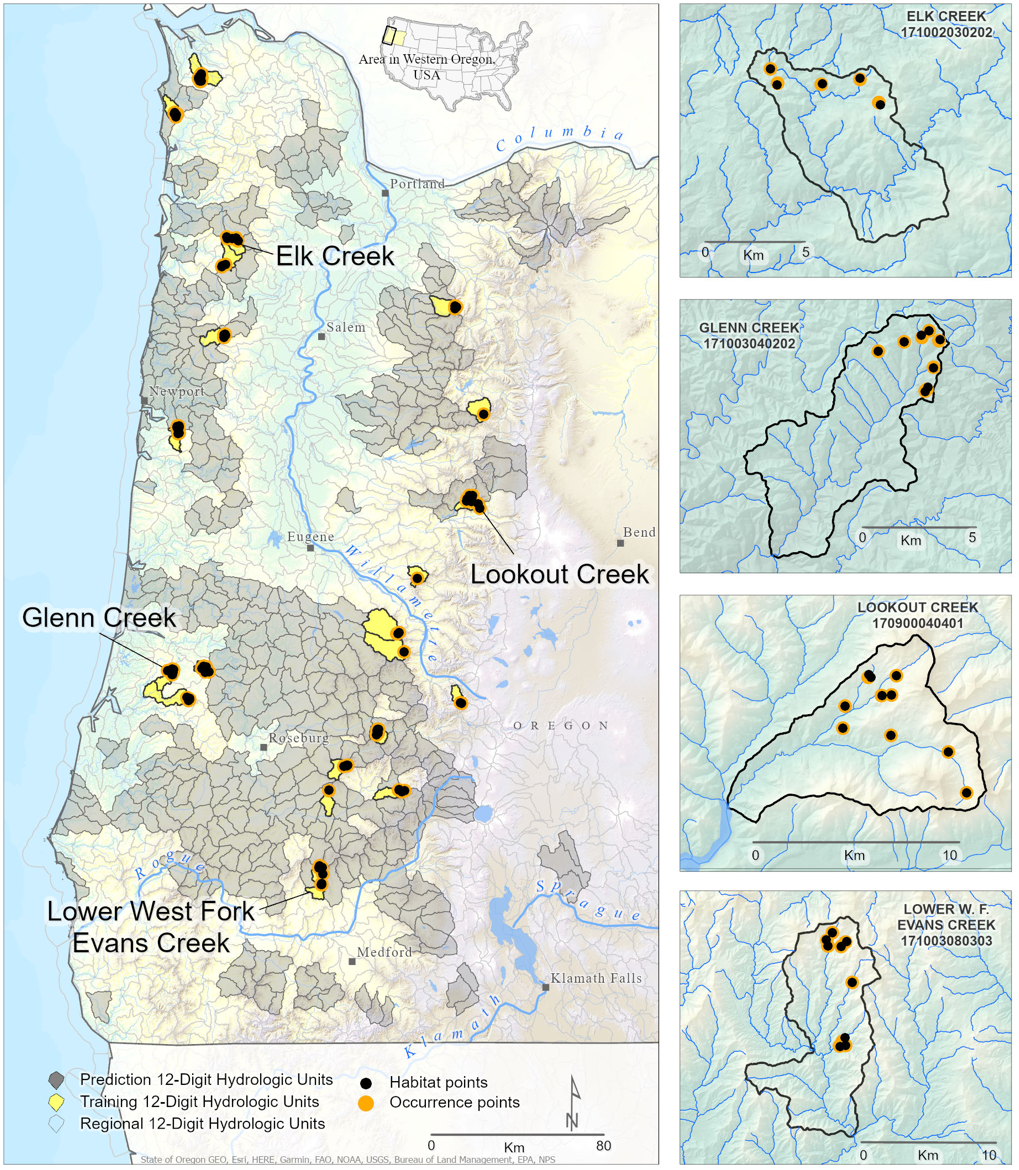


Figure S3. Study area map. The prediction domain for this study is the HUC12s in the study area in western Oregon that has complete LiDAR-derived hydrography uploaded to the NHD (USGS 2021) on 1 September 2021. Map was generated with ArcGIS Pro 3.0.2.


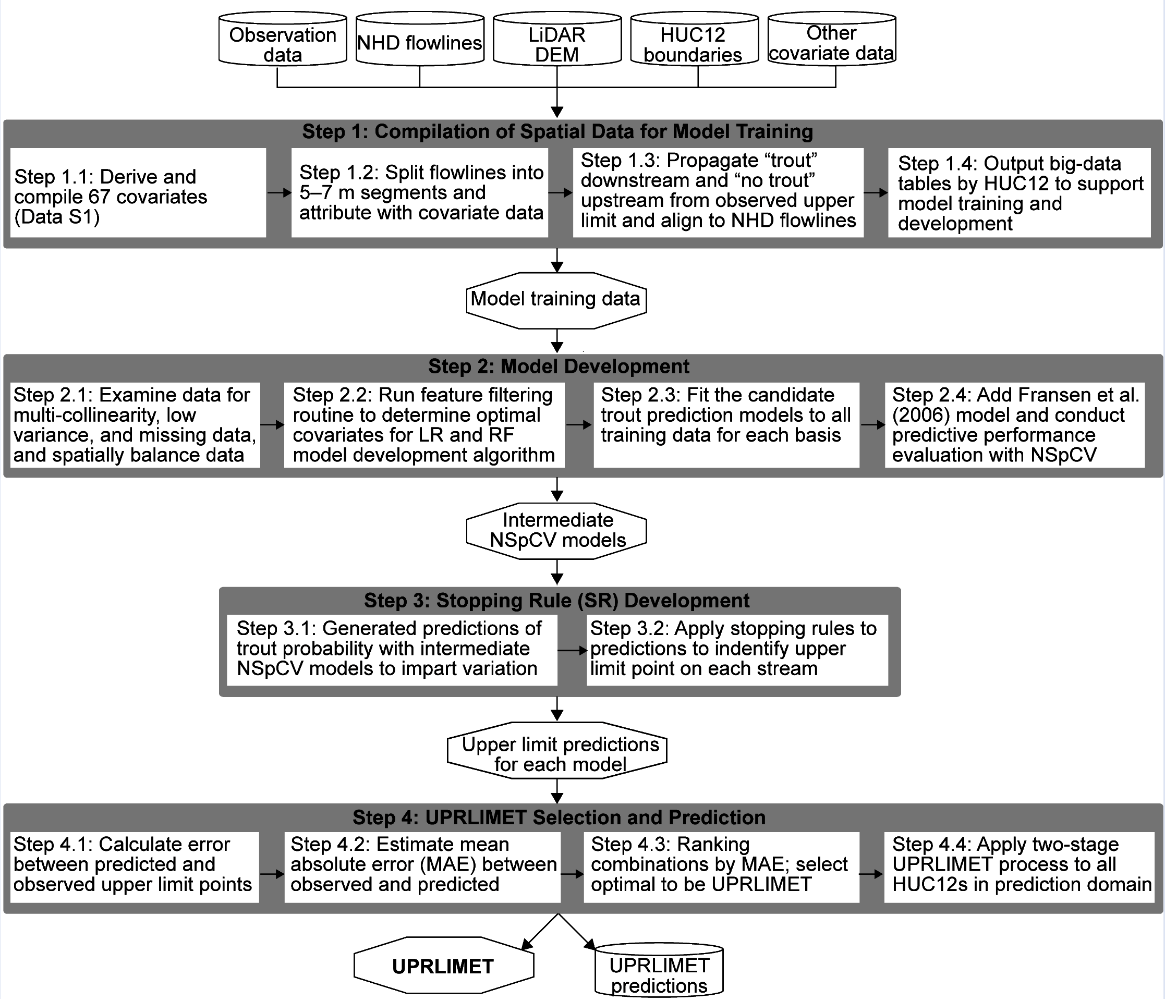


Figure S4. UPRLIMET development workflow depicting the four-step process taken to develop models, select UPRLIMET from the candidate models, and conduct UPRLIMET predictions across all HUC12s in the prediction domain. The culmination of the process is both the UPRLIMET model and predictions from UPRLIMET.

**Data Legends**

Data S1. Snapshot of table of 67 potential environmental predictor variables table. Shortname provides the variable name crosswalk to other tables (e.g., Data S8). Descriptive Name provides an intuitive name for the variable. Scale refers to the scale a given variable is characterized over. Local indicates a variable that is parameterized based on its condition at that location. Patch indicates a variable that is parameterized based on conditions broader than the local area of the reach. Parameterization indicates how data were summarized based on the scale. Ecological component refers to the dominant ecological component(s) influenced or represented by each variable. Ecological components were determined based on components known to be influential to upper limit of trout distributions. For example, several forest cover variables were represented, but a biological component is not specified because these variables were summarized for the upstream drainage area of each given point, that captures both upland and riparian areas. Given the mixing of upland and riparian, the size of the patch is too broad to adequately characterize variation from localized influence that the riparian zone has to a stream. Three general categories of ecological components are represented: streamflow (SF), connectivity (C), and biological (B). Most variables fell under the streamflow component because many of the hydro-topographic conditions that influence streamflow can be derived from a DEM, and DEMs were one of the few datasets available over the entire study area. Expected effect on trout occurrence indicates how increasing values of a variable are expected to relate to trout occurrence with the expected effect to be either positive or negative. Envelope indicates that the trout response to an increase in the variable is not always positive or negative across the full range of variable expression. Control variable indicates a variable that was included to characterize a large spatial extent environmental factors that likely influence upper limit of trout and how other covariates influence upper limit of trout. Derivation method indicates how the variable was derived from its source if source data were not used directly. Source resolution indicates the resolution of the source data for the variable. Geo-data source points to the internet location to download raw source data. Reference refers to the reference that describes the derivation of a given variable.


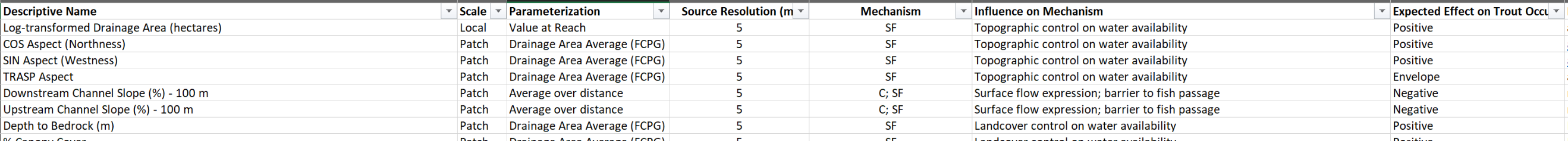


Data S2. Snapshot of candidate models for predicting upper limit of trout. This table offers an index of the model names with cross reference to the sub-model, model development algorithm, and training data types. The description column provides a brief explanation of what the model is for added clarification.


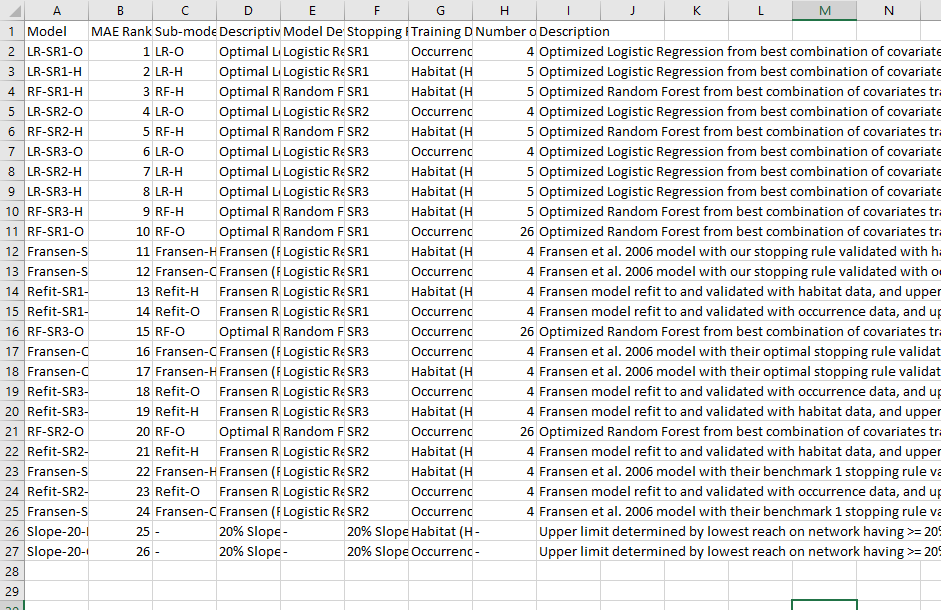


Data S3. Snapshot of error limits with confidence interval bars. Tabular output of the error metrics provided to assess the difference between predicted and observed upper limit for each Model. Upper and lower capture the 95% bootstrapped confidence interval and the Est. is the estimated median of the metric specified in the Metric column. Mean is mean difference, standard deviation is the standard deviation of differences, RMSE is root mean squared error, and MAE is mean absolute error, all in units of meters (m). Score is the ranking of the given model relative to the other models for a given metric. Note that MAE was used for selection and the other metrics are provided for context.


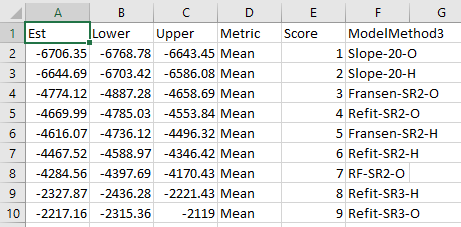


Data S4. Snapshot of covariates and filtering scores by model and training data type. This tabulates the ordering of covariates in ranked in descending order of importance by the Boruta filtering method. The Permutation and minimum joint mutual information maximization (JMIM) filter scores are provided for additional context and demonstrates the variation of importance by filter type. Feature names can be cross referenced with short names in Data S1for additional detail. LR, RF, Fransen, and Refit columns identify which variables are selected for the respective sub-models. The asterisk (*) identifies the covariates used in UPRLIMET for the purpose of comparison. Note that covariates from Fransen et al.^1^ are in the top 10, but these results indicate that they may not be best possible combination of covariates for maximizing prediction performance.


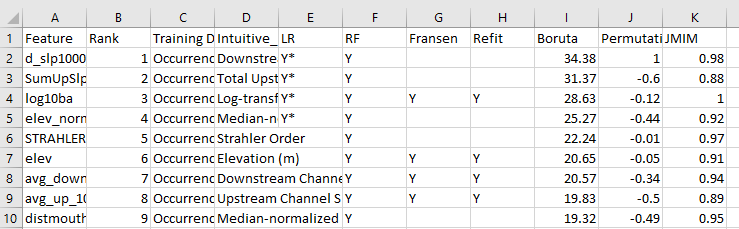


Data S5. Snapshot of trout probability model parameters. This tabulates the key parameters used for each sub-model developed in this study. Dynamic parameters are those that vary, such as coefficient values, whereas static parameters are those that are consistent for each model development algorithm type.


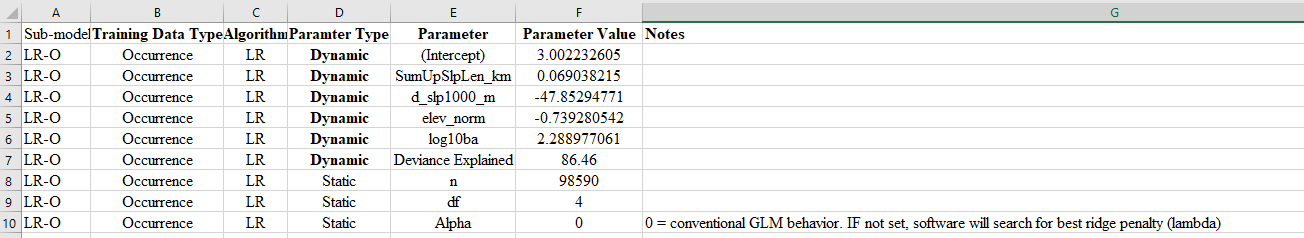


Data S6. Snapshot of model predictive performance. This tabulates the model predictive performance estimates of each for each sub-model and metric specified in the Performance Metric column and is produced in Step 2.4. of the model framework, assuming a class decision boundary at probability of 50%. These were used internally to understand how the sub-models were performing relative to each other. The columns labeled lwr and upr provide the bootstrapped 95% confidence interval around the estimated median (med) Accuracy is balanced accuracy, ROC-AUC is receiver operator characteristic area under the curve, NPV is negative predictive value, PPV is positive predictive value, PR-AUC is the precision recall area under the curve, CE is classification error (%), Brier is the Brier index, and MCC is Matthews Correlation Coefficient^2^. Fransen-Optimal models from Fransen et al.^1^ do not produce estimates of Brier, ROC-AUC, or PR-AUC because we only evaluated its classification accuracy in terms of binomial classes, and the aforementioned metrics require probabilities.


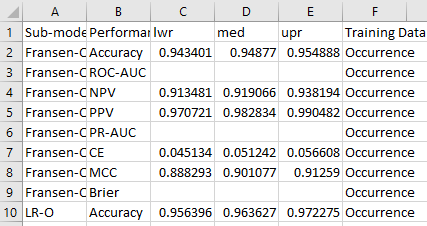


Data S7. Snapshot of guiding questions that drove development and selection of the potential models for predicting upper limit of trout (Data S2). With a few exceptions, questions were designed to produce a yes or no response. ‘Evaluation Process’ describes the process for addressing the question, ‘Evidence’ describes the result from the ‘Evaluation Process’ that would result in a ‘yes’ to the question. The ‘Sub-model’ describes which sub-models are evaluated to address the question, and the ‘Model’ describes which models were evaluated to address the question.


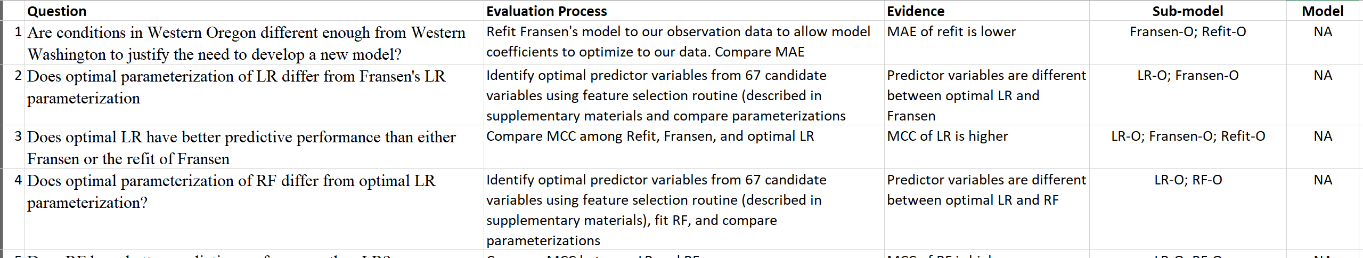


Data S8. Snapshot of MCC scores from feature selection. Matthew Correlation Coefficient (MCC)^2^ estimates with upper and lower 95% confidence intervals. In all cases, the covariate selections are drawn from the covariate importance list associated with the Boruta filtering method (Data S4). The Optimal column identifies which covariate combination resulted in the highest median MCC for a given model development algorithm and training data type combination, and subsequently defined the parameterization of the optimal sub-models for LR-H, LR-O, RF-H, and RF-O given LR=logistic regression, RF=random forest, H=habitat source data, and O=occurrence source data.


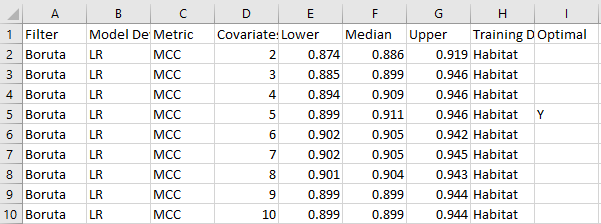


**Supplementary Methods**

**Model Framework**

An enhanced detailing of the four-step model framework summarized in the manuscript is described below and depicted in Fig. S4.

***Step 1 – Compilation of Spatial Data for Model Training***

In this step, a spatial database was compiled for each of the 21 HUC12s in the training domain with predictor covariates and the response variable. This step used Python ver. 3.7.4^3^ with significant dependencies on ArcGIS Pro (Ver 2.8, ESRI, Redlands, CA) to assemble the data necessary to support development of 26 candidate models for predicting the upper limit of trout through the following steps:

**1.1** For the 21 HUC12s in the training domain, we compiled 67 potential hydro-topographic variables (Data S1) selected to represent factors that relate to the following three general categories of ecological components that relate to the distribution of the upper limit of trout: streamflow (SF), connectivity (C), and biological (B). Variables included 5m resolution topo-hydrographic (e.g., channel slope, drainage area, surfacer roughness, etc.) metrics derived from 5m resolution LiDAR DEMs^4^, and 800m resolution climate (e.g., precipitation, air temperature) data for the 2017 calendar year as well as the 30-year climate normal period^5,6^. A major constraint on data inclusion was that it be available continuously across the entire spatial extent of the study area and hydrography, which had the effect of limiting incorporation of stream temperature from NorWest^7^ as well as other potentially important biotic and abiotic drivers not represented. Most variables fall under the streamflow ecological component because many hydro-topographic conditions that influence streamflow can be derived from a DEM and DEMs were readily available for the entire study area.

Variables were characterized in two scale categories, local and patch scale. Local refers to variables that represent conditions in the immediate vicinity of the point or reach of interest. Patch indicates a variable that is summarized for the reach of interest based on conditions broader than the local area of the reach. The most common patch scale parameterization was the drainage area weighted average of the variable as estimated with flow conditioned parameter grids (FCPGs)^8^. Other patch scale variables were characterized over different spatial extents or upland (e.g., Topographic Position Index) and different spatial extents of upstream and downstream (e.g., Slope over 100 m) as specified in Data S1. Dominant Ecological Component assignment for a given variable was determined by the variable itself, scale category, and size of patch, if applicable. For example, several forest cover patch scale variables are included in the table, but the presence of the stream-forest interaction zone did not warrant identifying a biological influence component because of the size of the patch. Patch condition was summarized over the entire upstream drainage area which captures both upland and riparian areas, and the influence was ascertained to be too broad to capture the more localized influence from just the riparian zone.

**1.2** LiDAR-derived NHD flowlines representing the stream network were then aligned to the 5m resolution DEM described in 1.1 and split the network into 5 to 7m reaches to ensure spatial agreement with the environmental prediction variables. Reach length varied depending how a given flowline crossed the corresponding DEM grid cells (7 m along diagonals). The 67 predictor variables were then indexed to the individual reaches. Reaches were then simplified to points representing the geographic center of the reach. Hereafter, points, and reaches are used synonymously, reflecting the fact that modeling occurs on points, but the points are representative of the reach.

**1.3** Occurrence (O) and habitat (H) observation data representing the upper limit of trout were aligned to the 5-7m reaches to support the development of binomial classification models in step 2. “trout” and “no trout” classifications were propagated downstream and upstream of the upper limit point, respectively, following the process described in Fransen et al*.*^1^, under the assumption that conditions downstream of an observed upper limit location are suitable for trout presence.

**1.4** Spatial data tables containing 67 covariates and the corresponding binomial response variable corresponding with the “trout” or “no trout” classifications were exported from Python to facilitate model development in step 2. Steps 1.1 and 1.2 were also used to generate spatial data for each HUC12 in the prediction domain (Fig. S2) to facilitate prediction with UPRLIMET in Step 4.4.

***Step 2 – Model Development***

This step develops the trout presence models in the R environment^8^ primarily using mlr3 framework for developing and testing machine-learning models^10^.

**2.1** Model training data were first examined for missingness as most modeling algorithms are not designed to internally handle missingness. Training data with missing data in any of the 67 predictor covariates were removed. Removal was chosen instead of interpolation, imputation, or simulated because the training dataset was very large at nearly 100,000 points. Data were also examined for low variation in covariates and those not expected to exhibit low variation were flagged for removal (none were identified). Categorical variables, and variables confined to the unit circle, such as aspect (and its derivatives) were expected to exhibit low variation. Only those variables where low variance was expected were identified, so none of the 67 candidate covariates were removed.

Model training data were then examined for multi-collinearity in R, by creating a design matrix of all 67 covariates and conducting a pairwise correlation analysis on each of the parameters (i.e., covariates) in the design matrix. The objective of the correlation analysis is to remove redundant variables to minimize data dimensionality and thus processing time spent examining all possible combinations of variables. The topic of multi-collinearity is covered in more detail in Step 2.3 under model delineation. Data were examined for unexpectedly large correlations. Only perfectly correlated (correlation = 1) were flagged for immediate removal. If correlation was less than one, we interpreted that to mean that there are locations on the stream network where the two highly correlated covariates do not vary to the same degree. Since the objective of this work was to isolate where on the stream network fish end, and that location ends up being a relatively small proportion of the overall network, we were hesitant to remove potentially useful information for the model. Furthermore, the overarching objective of this work is to produce the best prediction model from the data available, so the emphasis was more on predictive power than on process explanation. The latter point is especially key when considering the implementation of the Random Forest model development algorithm because Random Forest can make use of covariates that are weakly correlated to the response.

Data then underwent spatial balancing using undersampling of the majority class (whichever class is more frequent) to ensure equal numbers of ‘trout’ and ‘no trout’ training observations appear in each of the 21 HUC12s in the training domain. Undersampling randomly identifies points to be removed until class sizes (i.e., “trout” and “no trout”) are equal. We chose to use undersampling because the dataset was of sufficient volume that removing some data was assumed to have minimal influence on model performance and avoids the bias that can occur in estimating error and predictive performance when duplicate data is present. Spatial balancing ensures subsequent prediction models are fit such that 50% probability corresponds with the decision boundary between the positive (*i.e.,* “trout”) and negative class (*i.e.,* “no trout”). Class imbalances can result in models with a decision boundary other than 50%. This was a challenge that Fransen et al.^7^ overcame by searching for a different cut point because they had so many more positive class observations in their training data. We termed this process spatial balancing because balancing was done on a HUC12 basis instead of randomly selecting points from the whole dataset to achieve a balanced between the positive and negative classes. Simply employing balancing may have resulted in some HUC12s where the decision boundary between classes does not center on 50%.

**2.2**  Optimal combinations of covariates were selected for each combination of model development algorithm (*i.e.* logistic regression (LR) and Random Forest (RF)) and training data from the two different source types (*i.e.* O and H), using three different feature filtering methods (described later) to rank each of the 67 candidate covariates in terms of model importance. Feature filtering is a single-step method for identifying and ranking covariates with respect to the response in effort to reduce model dimensionality. Dimensionality reduction is desirable because unnecessarily high dimensionality (*i.e.,* more covariates than are necessary) can result in long processing times, produce large and complex RF models, increase the likelihood of overfitting in LR (when using conventional GLM), and can reduce model predictive performance when using either RF or LR algorithms^11^. Feature filtering is less computationally intense alternative to feature selection with cross-validation because each filter is run only once for each model-basis combination, whereas feature selection would have to be incorporated into the Nested Spatial Cross Validation (NSpCV) routine described in 2.4 to identify the covariate combination that maximizes predictive performance, substantially increasing the number of model fits for each model-basis^12^. Feature-filtering algorithms vary by prioritization approaches and may result in vastly different variable importance’s rankings. We examined three different filtering approaches Boruta, Joint Mutual Information Maximization (JMIM), and Permutation because these filters that have been shown to be effective in machine-learning applications^12,13,14^. The Boruta filter uses repeat fits of the Random Forest algorithm with all permutations of the variables and ranks importance by the frequency in which a variable is determined to be important to the model over all permutations. JMIM looks for variables that are complementary to already selected variables. The permutation method, as implemented in the MLR3 package uses Monte Carlo iterations to test all possible permutations of variables by fitting classification a regression tree (CART) model and ranking importance by how much the removal of a variable increased classification error compared to a CART with the variable included. Permutation is the most computationally intensive of the three algorithms.

After feature filtering, LR and RF models with 2 to 67 covariates (with covariates being added sequentially in descending order of importance (Data S4)) were fit to a spatially balanced 15% subsample of training. 15% was chosen to reduce processing time of this highly iterative process. Predictive performance was evaluated with the Matthews Correlation Coefficient (MCC)^2^ using a conventional 5-fold cross validation routine. We used MCC because it characterizes classification accuracy as the geometric mean of specificity and sensitivity, has been shown to be robust to class imbalances, and accounts for asymmetry in model specificity and sensitivity^15^. The optimal covariate combination for each model development algorithm and training data type was selected based on the lowest median MCC (Data S5). Only the results from the covariate combinations associated with the Boruta filter method (Data S4) are presented because regardless of model development algorithm or training data type, Boruta filtering produced covariate combinations that reached peak MCC with fewer features than the other two filtering methods tested.

**2.3** Eight different sub-models (Data S2) were developed to examine specific questions we developed internally to guide the process (Data S7). We implemented Fransen’s optimal 4-covariate model^1^ (here after the optimal Fransen model) to provide a reference for comparison. Fransen’s model is important for this comparison because it provides a regionally relevant benchmark for comparison. The model was developed using observation data in western Washington and GIS-derived variables from 10 m DEMs. Since the Fransen model^1^ was already developed and did not need to be fit to our data, we simply wrote manual function in R with the published model parameters. In the evaluation process described in the next step, optimal Fransen model^1^ effectively becomes two additional sub-models (for a total of eight), because it is tested against both O and H training data. The six remaining sub-models were composed of three sub-models fit to the O training data, and three sub-models fit to the H training data. The overarching three sub-model types are optimal Random Forest (RF), optimal Logistic Regression (LR), and the refit of Fransen (hereafter labeled Refit) and while distinct from each other are intended to provide a point of comparison between sub-models fit to O and sub-models fit to H data. For example, the RF-O (optimal RF fit to occurrence data) can be compared directly to RF-H (optimal RF fit to habitat data) to understand if models fit to O provides different prediction potential than models fit to H data. The optimal sub-models were designed to produce the best possible prediction models given the training data and 67 covariates. The Refit sub-models were designed with the intent of providing a direct comparison to the Fransen model^1^. If the Refit model performs better than the Fransen model^1^, then it would provide evidence that is likely a complex mixture of the improved stream network hydrography used in our study, and the improvement gain offered by re-calibrating Fransen’s model^1^ to the western Oregon prediction domain. Alternately, if the Refit model performs worse than Fransen’s model^1^, then it likely indicates that the NSpCV routine is overly conservative since the Refit model is optimized to the western Oregon landscape. The LR and RF algorithms are described in more detail in the paragraphs below.

RF and LR model algorithms were chosen owing to their previous usage in related stream-modeling applications^1,16^. RF is a machine learning model algorithm that uses an ensemble of weakly correlated decision trees to ascribe a response classification to a combination of covariates, is inherently accounts for interactions and is robust to multi-collinearity among covariates^17^. To minimize the potential for overfitting, we used a high number of trees (1500), set the resampling to bootstrap, tuned the ‘mtry’ parameter using a nested loop, and established a minimum node size of ten. We used the Ranger^18^ implementation of random forest in R because it uses parallelization to reduce processing times.

LR is a form of a generalized linear model (GLM) development algorithm that fits a log-linear relationship between covariates and response data using a logit function. In this application, the binomial response data are the “trout” and “no trout” classifications assigned to each point. LR produces optimal parameter values when predictor covariate data are centered and scaled so that disproportionately large variances in a particular covariate relative to another does not overly influence the model fit. Additionally, LR assumes covariates are independent of each other. When highly colinear covariates are present, the standard error estimates derived from the LR model are not representative of actual conditions. Although some of the 67 predictor variables exhibit collinearity, we chose not to filter them to ensure RF and LR algorithms were presented with the same data. The cross-validation routine we assembled to evaluate prediction error does not rely on internal model estimates of standard error, so the concern regarding the representativeness of model standard errors is not a concern for our application. To develop the LR sub-models, we used the Glmnet implementation in R^19^. In the Glmnet parameterization, we allowed the model to internally center and scale the predictor covariate data. The relaxation parameter was turned off and the ridge penalty was set to 0 to force the Glmnet to parameterize like the conventional logistic regression used in Fransen et al.^1^

**2.4**  The predictive performance of the eight sub-models were then evaluated with a Nested Spatial Cross Validation (NSpCV) routine composed of 21 spatial groups using of 5 repeats of a 5-fold CV routine. This routine produced 25 intermediate sub-models fit to randomly selected subsets of spatial groupings (described below) of the training data respective to each of the eight sub-models. The optimal Fransen model^1^ is tested with this routine, using the identical subsets of spatial groups of the other models in effort to test predictive performance in a comparative way, but intermediate sub-models are not produced because it was developed outside of this study on independent data. NSpCV was chosen over the more conventional approach of splitting data into training, validation, and test sets because it allows for an unbiased evaluation of predictive performance for models with tuned hyperparameters^8^ and spatial CV has been shown to produce conservative estimations of predictive performance when data are likely to be spatially correlated^20,21^. With conventional data splitting there is a possibility for spurious selection of a test data subset that does not adequately represent the spatial variability in the training set, which may lead to an overly optimistic or overly pessimistic estimation of predictive performance that does not represent the average performance across the prediction domain. Results are presented in Data S7. Additional detail on NSpCV is provided in explanatory paragraphs below.

The NSpCV routine was implemented with the mlr3spatiotempcv package in R^22^. It works by assigning training data in each of 21 HUC12s and to one spatial group for each HUC12, under the assumption that conditions within a HUC12 are more similar (*i.e.,* correlated) than conditions among HUC12s. The routine then fits intermediate sub-models to the training data using a 5-repeat 5-fold resampling routine whereby the 21 spatial groups are randomly split into five folds. An intermediate sub-model is fit to training data in 4 of the 5 folds and tested against the one hold-out fold and predictive performance estimates in terms of MCC, Negative Predictive Value (NPV), Positive Predictive Value (PPV), Accuracy, Receiver Operator Characteristic Area Under the Curve (AUC), and Precision-Recall Area Under the Curve (PRAUC), are produced. This is repeated until each fold has been tested. The 5-fold routine is then repeated four more times (*i.e.,* 5-repeat) and the reshuffling of spatial grouping assignment to each of the folds induces variation to provide a representation of the range of variability in predictive performance. Although more repeats would likely better represent the full range of performance variability, we were limited to five repeats by our computational capacity. Completion of the NSpCV routine resulted in 25 intermediate sub-models, and 25 estimates of each of the predictive performance metrics for each sub-model. Boot-strapped confidence intervals and medians were calculated for each metric with 10,000 bootstraps the data using the boot function in R. Randomization seeds were utilized for the NSpCV process to ensure that all intermediate sub-models used the same data subsets for training and testing.

**Step 3 – *Stopping* Rule Development**

The purpose of stopping rules (SRs) as described in Fransen et al.^1^ is to classify stream reaches into binomial “trout”, ‘no trout’ classes based on the predicted trout presence probability at each reach while accounting for upstream and downstream conditions such that only a single point on a stream is the upper limit. We developed and tested three different SRs. SR1 is a variation on Fransen’s optimal rule^1^ using the same trigger size (1) and block size (184) but applies a rolling average to smooth the predicted probabilities. Probability threshold (i.e., cut point) for the trigger is set to 50% and a search was conducted for the presence of a slope of 30% or more between the initial trigger (the lowest point with ≥ 50% probability) and any potential upstream triggers to prevent moving the upper limit upstream of a waterfall (Fig. S4). SR2 uses the lowest point on the stream having a probability ≥ 50%, which is analogous to Fransen’s benchmark 1 stopping rule, and SR3 is the optimal stopping rule described in Fransen et al.^1^.

**3.1** For each of the eight sub-models (Step 2.3; Data S2), we generated predictions of trout presence probability on the flowlines for each of the 21 HUC12s having observation data using the 25 intermediate sub-models produced during the NSpCV routine in Step 2.4. The 25 intermediate sub-models were used for this step to continue the process of evaluating predictive performance on subsets of the training data. For a given intermediate sub-model, predictions were only produced for the approximately 20% of HUC12s that were held out of the training dataset that was used to train that intermediate sub-model. Note that two Fransen Optimal sub-models^1^ (i.e., Fransen-O, Fransen-H; Data S2) are full parameterizations so there are no intermediate sub-models to apply. However, for the sake of paired comparisons, the Fransen Optimal sub-models^1^ were applied to identical subsets of the held out HUC12s for the other sub-models to facilitate paired comparison in step 4.

**3.2** For each of the eight sub-models in Step 3.1, each of the three stopping rules (SRs) described above were applied to each of the associated intermediate sub-model predictions to predict the upper limit of trout along the stream. This results in 24 sub-model and stopping rule combinations which are hereafter referred to as ‘models’ to simplify the narrative Stopping rules are applied by first identifying each combination of stream outlet and stream initiation point (as reflected in the flowlines), then applying the stopping rule logic to all of the reaches between the two points. The observed upper limit point between the outlet and inception point are also identified for error analysis in step 4 below. Applying the SRs in this manner results in an upper limit of trout prediction for each initiation point, although many initiation points tend to share the same upper limit of trout location.

An additional upper limit of trout prediction method that identifies the upper limit at the lowest point on the stream where channel slope is greater than or equal to 20% over a 20 m run of stream was applied to provide a commonly understood reference to compare to the results of the other 24 models. The method is commonly applied in Oregon when observation data are not available to inform the upper limit of fish^23^. While we refer to this method as two separate models (*i.e.,* 20%-O, 20%-H; Data S2) for the sake of simplifying comparisons with the other 24 models, it is more like a stopping rule. However, we disambiguate it from stopping rule because it requires channel slope information instead of probability of trout presence. Upper limit predictions using the 20% slope method were applied iteratively to the same subsets of held out HUC12s as the Fransen Optimal sub-models^1^ in 3.1 above so that resulting predictions would be properly pooled for comparison to the other models. To find the upper limit with 20% slope, each stream inception point, and outlet are identified and all reaches are sequentially arranged from the outlet to inception. The stream reach immediately downstream of the reach having a 20% slope over 20 m is determined to be the upper limit. The observed upper limit point between the outlet and inception point are also identified for error analysis in step 4 below. Applying the 20% method in this manner results in an upper limit of trout prediction for each initiation point, although many initiation points tend to share the same upper limit of trout location. For all stopping rules and methods, the upper limit search space was limited to stream reaches having drainage areas greater than or equal to 0.2428 ha (60 acres), following the logic employed by Oregon Forest Practices Act for modeling in the Coast Range when water or pool depth are unknown [23]. The Coast Range rule was applied uniformly across the study area for consistency. Consequently, if the predicted upper limit point occurs on a reach having a drainage area less than 0.2428, the upper limit point is moved downstream to the nearest reach having a drainage area greater than or equal to 0.2428 ha.

**Step 4 – UPRLIMET Selection and Prediction.**

**4.1**  For each of the 26 models (Data S2), linear error in units of meters was calculated between observed predicted upper limit locations, where observed is the associated O or H data, depending on the model specification. Since the alignment process described in 1.2 above calculates the linear stream distance between any given point on the stream and the stream outlet (typically bottom edge of HUC12 boundary), the error was calculated as the differences between distance to outlet for the predicted and observed points and aggregated into a single data table.

**4.2** For each of the 26 models, estimate four different summary error metrics from the linear errors in 4.1 above. Mean Absolute Error (MAE; Eq. 1), Root Mean Squared Error (RMSE; *i.e.,* square root of the mean of the squared linear errors), bias (*i.e.,* mean of linear errors), and standard deviation. MAE was selected as the metric for choosing the best model for predicting the upper limit of trout because MAE is an intuitive summary metric that accounts for bias and variance but does not have the same sensitivity to large variances as RMSE. RMSE, bias, and standard deviation are included because these are other measures of error readers may be familiar with and each characterizes the data differently. RMSE is unsigned, is a composite measure of bias and variance, and can be heavily skewed by large variances. Bias is signed, so it can inform on general trend in terms of directionality to provide measure of whether the model over or under predicts. Standard deviation is a commonly used measure of variation and provides a sense of how error varies about the mean. Boot-strapped 95% confidence intervals and median was calculated for each metric with 10,000 bootstraps of the error estimates using the boot function in R. Resulting error metrics are presented in Data S3.

$${MAE}_{s_{r}}= \frac{\sum_{i=1}^{{n_{s}}_{r}} |y_{i_{s_{r}}}-x_{i_{s_{r}}}|}{n_{s_{r}}}$$

Eq 1: Mean absolute error [MAE]—Where *i* is an observed upper limit from *n* upper limit observations in subset *s of* the training data where *s* corresponds with each of 5-folds within each *r* of 5 repeats of the NSpCV routine. *y* is the linear stream distance (m) of the observed upper limit point from the HUC12 outlet for a given model, and *x* is the linear stream distance (m) of the predicted upper limit point and the difference between *y* and *x* represents error.

**4.3** Median MAE for each model from 4.2 above was ranked in ascending order. The model number one ranked model was selected to become UPRLIMET.

**4.4** To prepare stream networks for 383 HUC12s within the prediction domain, steps 1.1 and 1.2 were applied to generate 5-7m stream reaches and the predictor variables required for UPRLIMET along the stream networks. The two-stage UPRLIMET model (Fig. S2) was then applied to the stream network to first predict the probability of trout presence at each stream reach (Stage 1) and then apply the stopping rule (Stage 2) to predict discrete upper limit locations from the predictions.

**Comparison of trout distribution datasets with UPRLIMET**

We compared UPRLIMET to two trout distribution datasets, including the Coastal Cutthroat Trout Interagency Commission (ICCT) and the Oregon Department of Forest (ODF) datasets. For the ICCT dataset, Coastal Cutthroat Trout observation data were gathered from 90 state and federal agencies, Tribal nations, municipalities, universities, individuals, and non-governmental organizations throughout the US portion of the sub-species geographic distribution (northern California to south central Alaska). Observation data were compiled into a single data-base and aligned with stream hydrography or, if documentation occurred upstream of the existing hydrography, the stream-path was extended manually. These data were intended to document the occurrence of CCT to support a range-wide assessment of the sub-species by identifying occupancy trends and support agency monitoring efforts. As such the database includes associated records to trace the provenance of the collection. The data that support the findings of this study are openly available at <http://doi.org/10.7923/Z5ZN-7219>. For the ODF dataset, the Oregon statewide streams dataset^24^ provides a stream layer, including fish presence data for the state of Oregon. The statewide streams information says that “Forest practice rules require tree retention along streambanks where and fish and domestic water use supplies are involved. Operators must protect soils, fish and wildlife habitat, and water quality. Protecting these stream sides promote mature forest conditions, helps maintain fish habitat and prevents sediments from entering the stream.”

References

1. Fransen, B. R., Duke, S. D., McWethy, L. G., Walter, J. K., & Bilby, R. E. (2006). A logistic regression model for predicting the upstream extent of fish occurrence based on geographical information systems data. North American Journal of Fisheries Management, 26(4), 960-975.
2. Matthews, B. W. (1975). Comparison of the predicted and observed secondary structure of T4 phage lysozyme. Biochimica et Biophysica Acta (BBA)-Protein Structure, 405(2), 442-451.
3. Van Rossum, G., & Drake, F. L. (2009). Python 3 Reference Manual. Scotts Valley, CA: CreateSpace.
4. DOGAMI. LIDAR Digital Terrain Model Mosaic. Scale Not Given. Not Dated. https://gis.dogami.oregon.gov/arcgis/rest/services/LiDAR/DIGITAL_TERRAIN_MODEL_MOSAIC_HS/ImageServer (Accessed 13 May 2022).
5. Daly, C., & Bryant, K. (2013). The PRISM climate and weather system—an introduction. Corvallis, OR: PRISM climate group, 2.
6. PRISM. LIDAR Oregon State University PRISM Climate Group. Scale Not Given. Not Dated. https://prism.oregonstate.edu/. (Accessed August 2021).
7. Isaak, D. J., Wenger, S. J., Peterson, E. E., Ver Hoef, J. M., Nagel, D. E., Luce, C. H. & Parkes‐Payne, S. (2017). The NorWeST summer stream temperature model and scenarios for the western US: A crowd‐sourced database and new geospatial tools foster a user community and predict broad climate warming of rivers and streams. Water Resources Research, 53(11), 9181-9205.Barnhart, T.B., Sando, R., Siefken, S.A., McCarthy, P.M., and Rea, A.H., 2020, Flow-Conditioned Parameter Grid Tools: U.S. Geological Survey Software Release, DOI: <https://doi.org/10.5066/P9W8UZ47>.
8. Barnhart, T.B., Sando, R., Siefken, S.A., McCarthy, P.M., and Rea, A.H., 2020, Flow-Conditioned Parameter Grid Tools: U.S. Geological Survey Software Release, DOI: <https://doi.org/10.5066/P9W8UZ47>.
9. R Core Team (2013). R: A language and environment for statistical computing.
10. Lang, M., Binder, M., Richter, J., Schratz, P., Pfisterer, F., Coors, S., ... & Bischl, B. (2019). mlr3: A modern object-oriented machine learning framework in R. Journal of Open Source Software, 4(44), 1903.
11. Kirasich, K., Smith, T., & Sadler, B. Random forest vs logistic regression: binary classification for heterogeneous datasets. SMU Data Science Review, 1(3), 9. (2018).
12. Bommert, A., Sun, X., Bischl, B., Rahnenführer, J., & Lang, M. (2020). Benchmark for filter methods for feature selection in high-dimensional classification data. Computational Statistics & Data Analysis, 143, 106839.
13. Kursa, M. B., Rudnicki, W. R., & Kursa, M. M. B. (2020). Package ‘Boruta’.
14. Speiser, J. L., Miller, M. E., Tooze, J., & Ip, E. (2019). A comparison of random forest variable selection methods for classification prediction modeling. Expert systems with applications, 134, 93-101.
15. Chicco, D., Jurman, G. The advantages of the Matthews correlation coefficient (MCC) over F1 score and accuracy in binary classification evaluation. BMC Genomics 21, 6 (2020). [[https://doi.org/10.1186/s12864-019-6413-7https://doi.org/10.1186/s12864-019-6413-7](https://doi.org/10.1186/s12864-019-6413-7)](https://doi.org/10.1186/s12864-019-6413-7)
16. Jaeger, K. L., Sando, R., McShane, R. R., Dunham, J. B., Hockman-Wert, D. P., Kaiser, K. E., ... & Blasch, K. W. (2019). Probability of Streamflow Permanence Model (PROSPER): A spatially continuous model of annual streamflow permanence throughout the Pacific Northwest. Journal of Hydrology X, 2, 100005.
17. Breiman, L. (2001). Random forests. Machine learning, 45(1), 5-32.
18. Wright, M. N., & Ziegler, A. (2015). ranger: A fast implementation of random forests for high dimensional data in C++ and R. arXiv preprint arXiv:1508.04409.
19. Hastie, T., & Qian, J. (2014). Glmnet vignette. Retrieved June, 9(2016), 1-30*.*
20. Tsamardinos, I., Rakhshani, A., & Lagani, V. (2015). Performance-estimation properties of cross-validation-based protocols with simultaneous hyper-parameter optimization. International Journal on Artificial Intelligence Tools, 24(05), 1540023.
21. Roberts, D. R., Bahn, V., Ciuti, S., Boyce, M. S., Elith, J., Guillera‐Arroita, G., & Dormann, C. F. (2017). Cross‐validation strategies for data with temporal, spatial, hierarchical, or phylogenetic structure. Ecography, 40(8), 913–929.
22. Schratz, P., Becker, M., Lang, M., & Brenning, A. (2021). Mlr3spatiotempcv: Spatiotemporal resampling methods for machine learning in R. arXiv preprint arXiv:2110.12674.
23. Oregon Department of Forestry. Forest Practices administrative Rules and Forest Practices Act. 629 Forest Practices Administration. In effect January 1, 2021. https://www.oregon.gov/odf/Documents/workingforests/fpa-rule-book-2021.pdf (Accessed 16 July 2022).
24. Oregon Department of Forestry. Statewide streams. <https://www.oregon.gov/odf/aboutodf/pages/mapsdata.aspx> (Accessed 12 October 2021).
